# Pericyte-mediated intercellular crosstalk in renal fibrosis: regulatory mechanisms and intervention strategies

**DOI:** 10.3389/fphys.2026.1963705

**Published:** 2026-10-21

**Authors:** Lei Tang, Wei Qiao, Suyu Wang, Zhao Wang, Shaohong Zhang, Ling Liu, Wenbo Zhu

**Affiliations:** 1 Department of Chinese Medicine, Jiangsu College of Nursing, Huaian, China; 2 Department of Clinical Medicine, Jiangsu College of Nursing, Huaian, China; 3 Department of Gerontology, The Affiliated Huaian No.1 People’s Hospital of Nanjing Medical University, Huaian, China; 4 Jiangsu Province Engineering Research Center of Precise Prevention in the Digestive and Reproductive System Cancers, Jiangsu College of Nursing, Huaian, China

**Keywords:** engineered extracellular vesicle, intercellular crosstalk, mesenchymal stem cell therapy, pericyte, pericyte-to-myofibroblast transition, renal fibrosis

## Abstract

Chronic kidney disease continues to increase in global prevalence, with renal fibrosis serving as a major pathological driver of end-stage renal disease and imposing a substantial burden on global public health. Renal pericytes, essential mesenchymal cells distributed along capillary walls, have been redefined from mere structural vascular support cells to multidirectional communication hubs that coordinate homeostasis of the renal microenvironment. Under persistent injurious stimuli, pericytes detach from capillary walls, migrate, and become activated, undergoing pericyte-to-myofibroblast transition and emerging as a predominant source of renal interstitial myofibroblasts. This process promotes capillary rarefaction and aberrant extracellular matrix deposition, thereby establishing a vicious cycle of “vascular loss–exacerbated hypoxia–progressive fibrosis”. Herein, the pericyte-mediated cellular interactome and its molecular regulatory mechanisms in renal fibrosis are systematically reviewed, with particular emphasis on the bidirectional communication of pericytes with vascular endothelial cells, renal tubular epithelial cells, immune cells (including macrophages, T lymphocytes, neutrophils, and mast cells), and fibroblasts; direct renal evidence for these interactions is explicitly distinguished from indirect or hypothetical mechanisms inferred from non-renal models. The roles of key signaling pathways, including Ang/Tie2, PDGF/PDGFRβ, TGF-β/Smad, Hippo-YAP/TAZ, Wnt/β-catenin, and Notch, in regulating these interactions are further elucidated, with clarification of the extent to which each pathway is pericyte-intrinsic or involves pericytes as one of several effector cell types. Additionally, recent advances in intervention strategies targeting pericyte-mediated crosstalk are critically appraised according to the directness of pericyte targeting and the strength of the supporting evidence, encompassing mesenchymal stem cell therapy, engineered extracellular vesicles, nanomedicine-based drug delivery systems, and natural pharmaceutical therapies. Current evidence remains constrained by the lack of pericyte-specific markers, the reliance on rodent models, and the predominantly preclinical stage of available interventions; nevertheless, a comprehensive understanding of the pericyte-mediated cellular interactome may provide an important theoretical foundation and candidate therapeutic targets for the prevention and treatment of renal fibrosis.

## 1 Introduction

Chronic kidney disease (CKD) is increasing in prevalence worldwide, with end-stage renal disease (ESRD) imposing a substantial medical burden on patients and society. Renal fibrosis represents a major pathological process underlying the progression of CKD to ESRD (Liu, 2011). Previous studies have predominantly focused on aberrant activation of individual cell types or dysregulation of isolated signaling pathways and have therefore failed to systematically elucidate the complex pathological networks and molecular interactions governing renal fibrosis. As a highly complex organ composed of diverse cell types, the kidney depends on coordinated intercellular communication to maintain its structural integrity and physiological homeostasis (Scheidereit et al., 2026). Under injury-induced stress, aberrant remodeling of intercellular communication networks constitutes an important initiating mechanism that drives the sequential pathological progression of renal fibrosis (He et al., 2024).

Renal pericytes are located on the abluminal aspect of the basement membrane of peritubular capillaries and constitute an integral component of the perivascular mesenchymal cell population, with capacities for vascular structural support, paracrine regulation, and multilineage differentiation. Under physiological conditions, pericytes maintain homeostasis of the renal microvascular network and interstitial microenvironment through intercellular crosstalk. Following sustained pathological insults, pericytes can dissociate from capillary walls and undergo aberrant activation, thereby contributing to inflammatory amplification (Chen et al., 2011), abnormal extracellular matrix (ECM) deposition (Yang et al., 2019), and impairment of the microvascular network (Kramann et al., 2017). Through advanced methodologies such as Single-cell RNA sequencing (scRNA-seq) and spatial transcriptomics, the important role of pericytes as hubs of renal intercellular communication has been increasingly elucidated, positioning pericyte-mediated cellular interactions as a prominent target in research on antifibrotic therapies for the kidney. This review systematically delineates the interactions between pericytes and diverse renal cell types, explicitly distinguishing direct kidney-specific evidence from indirect evidence and cross-organ extrapolations, and summarizes the molecular mechanisms through which these interactions regulate the initiation and progression of renal fibrosis, with clarification of which mechanisms are genuinely mediated by renal pericytes. Intervention strategies are critically appraised according to whether they directly target renal pericytes or indirectly modulate pericyte behavior, and current limitations and future research directions are highlighted, thereby providing a theoretical foundation for elucidating the mechanisms of renal fibrosis and for developing strategies for its prevention and treatment.

## 2 Methodology

### 2.1 Databases and search strategy

This review was conducted and reported in accordance with the 2020 PRISMA (Preferred Reporting Items for Systematic Reviews and Meta-Analyses) statement. A systematic search was conducted in six electronic databases (PubMed, Web of Science Core Collection, Google Scholar, ScienceDirect, China National Knowledge Infrastructure (CNKI), and SciFinder), with the search period ending on July 31, 2026. There was no restriction on the starting date of publication; only articles published in English or Chinese were considered as eligible research subjects. Additionally, the references of all included articles were manually screened, related reviews were performed, and other eligible studies were identified through citation tracking.

The search strategy combined controlled vocabulary with free-text terms from five key domains: (1) renal fibrosis, fibrosis in other tissues, and related renal pathological contexts; (2) pericyte biology and molecular identification; (3) pericyte-mediated intercellular interactions; (4) pericyte-associated signaling pathways; and (5) therapeutic interventions targeting pericyte-related mechanisms. Therefore, we selected five key terms: “renal fibrosis”, “pericytes”, “cell-cell interaction”, “signal pathway”, and “intervention strategy” to collect relevant literature, and analyzed according to the standards.

### 2.2 Study eligibility criteria

Studies were eligible if they: (1) addressed at least one predefined domain; (2) focused on pericytes in tissue fibrosis, particularly renal fibrosis and its pathological microenvironment; or (3) provided mechanistic, multi-omics, or clinically relevant evidence from *in vitro* experiments, animal models, human kidney tissues, or clinical samples.

Studies were excluded if they: (1) were conference abstracts, commentaries, letters, patents, book chapters, or duplicate publications; (2) had no direct association with fibrosis or pericyte biology; or (3) examined only non-pericyte-derived myofibroblasts without providing mechanistic insight or comparative relevance to pericytes; (4) had insufficient methodological detail or outcome data.

### 2.3 Study selection and data collection

All records retrieved from the databases were imported into EndNote 21 (Clarivate, Philadelphia, PA, USA), and any duplicate records were removed through a combination of automated deduplication and manual verification. Two investigators independently screened the titles and abstracts according to the eligibility criteria and then reviewed the full texts of potentially relevant studies. Disagreements were resolved through discussion or, when necessary, adjudication by a third investigator. For each eligible study, the first author, publication year, journal, study type, experimental model, interacting cell types, underlying mechanisms, interventions, principal findings, and limitations were extracted. Particular attention was given to lineage-tracing studies, single-cell or spatial multi-omics analysis, and *in vivo* functional validation, with careful evaluation of whether the findings directly supported intercellular communication mechanisms rather than merely showing cellular colocalization or concurrent phenotypic changes.

### 2.4 Literature search results

The study selection process is summarized in the PRISMA 2020 flow diagram (**Figure 1**). The initial database search yielded 18,130 records, comprising PubMed (n = 6,828), Web of Science (n = 4,867), ScienceDirect (n = 2,148), CNKI (n = 131), SciFinder (n = 876), and Google Scholar (n = 3,280). After the removal of 5,206 duplicate records, the titles and abstracts of the remaining 12,924 records were screened, and 12,602 records that were clearly irrelevant to the review scope were excluded. The full texts of the remaining 322 reports were sought for retrieval, and all 322 reports were successfully obtained and assessed for eligibility. Of these, 143 reports were excluded for the following reasons: ineligible publication type (conference abstract, commentary, letter, patent, or book chapter; n = 34); no direct association with fibrosis or pericyte biology (n = 51); exclusive focus on non-pericyte-derived myofibroblasts without mechanistic insight or comparative relevance to pericytes (n = 38); and insufficient methodological detail or outcome data (n = 20). Ultimately, 179 articles were included in this review.

**Figure 1:**
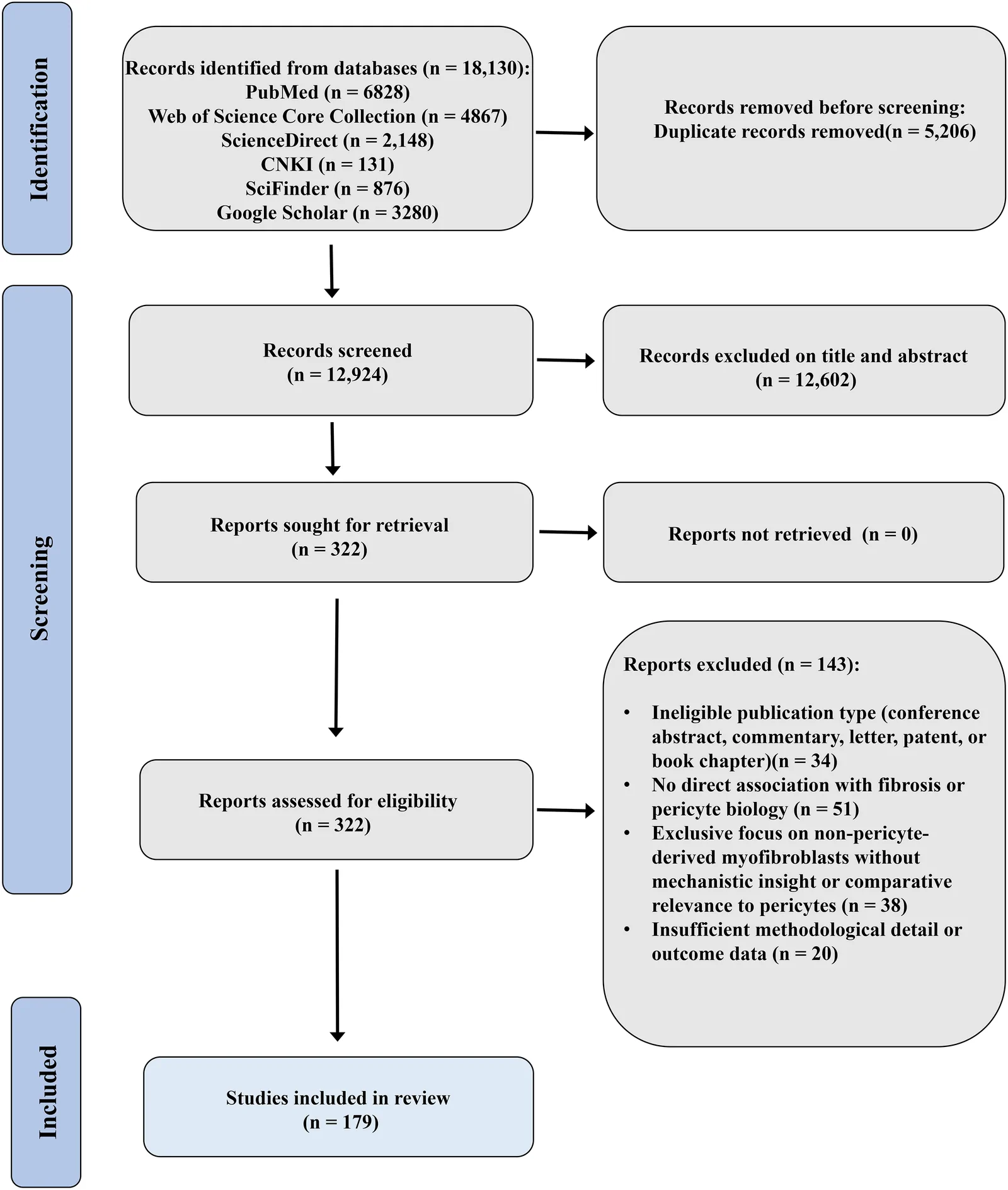
PRISMA flow diagram of study screening and selection.

The included literature comprised foundational studies defining pericyte biology, lineage relationships, and canonical profibrotic pathways, as well as recent *in vitro* and *in vivo* mechanistic studies of renal fibrosis, human renal transcriptomic and spatial omics analysis, clinical and observational studies, therapeutic intervention studies, and selected high-quality reviews. Taken together, this evidence base supports the thematic structure of this review, which encompasses pericyte biology, intercellular communication, signaling regulation, and therapeutic strategies.

## 3 Biology of pericytes

### 3.1 Definition and morphology of pericytes

In the early 19th century, the French scientist Charles-Marie Benjamin Rouget first identified a population of contractile cells associated with small blood vessels, which were termed Rouget cells. Subsequently, the German scientist Karl Wilhelm Zimmermann renamed these cells “pericytes” based on their perivascular anatomical location (Birbrair, 2018; Armulik et al., 2011). Pericytes are widely distributed along the abluminal surface of the endothelial cell (EC) basement membrane throughout the systemic microvasculature. They are characterized by a prominent oval nucleus and cytoplasmic processes that extend longitudinally along the microvascular axis and span several adjacent ECs (Attwell et al., 2016). These processes progressively branch and taper toward their distal ends to encircle the microvascular wall, thereby maintaining vascular stability through paracrine signaling and physical interactions with ECs and the basement membrane (Ayloo et al., 2022).

Pericyte morphology and vascular coverage vary according to the type of vascular bed and differentiation status. In the central nervous system, pericytes exhibit a characteristic flat, stellate morphology and are collectively embedded within astrocyte-derived vascular basement membranes, with a pericyte-to-endothelial cell ratio ranging from 1:1 to 1:3 (Drozd et al., 2025; Zheng et al., 2020). Pulmonary pericytes are fusiform or stellate, with an approximate pericyte-to-endothelial cell ratio of 1:7 to 1:9 (Wilson et al., 2018). *In vitro*, coronary microvascular pericytes adopt an antler-like morphology and possess slender filopodia (Nees et al., 2011). In the heart, the pericyte-to-endothelial cell ratio ranges from 1:2 to 1:3, making pericytes the second most abundant non-cardiomyocyte cell type after endothelial cells (Su et al., 2021). Hepatic stellate cells (HSCs) are regarded as liver-resident pericytes. These cells perform canonical pericyte functions, including vascular regulation and matrix homeostasis, while also possessing the liver-specific capacity to store vitamin A (Du et al., 2024). Located between hepatocyte layers and hepatic sinusoidal endothelial cells, HSCs display a distinctive stellate phenotype, with long cytoplasmic processes wrapped around hepatic sinusoids, and have a pericyte-to-endothelial cell ratio of approximately 1:8 to 1:10 (Li and Fan, 2023; Marcos and Correia-Gomes, 2016). Renal pericytes include tubular pericytes, podocytes and mesangial cells (Smith et al., 2012). These cells circumferentially line capillaries and form close junctions with ECs to maintain vascular network stability, modulate vasomotor tone and regulate vascular permeability. Renal pericytes are rounded and have a pericyte-to-endothelial cell ratio of approximately 1:2.5 (Zhang et al., 2020; Humphreys et al., 2010). **Table 1** summarizes the morphological and distributional features of pericytes across different tissues.

**Table 1:** Morphological features and distribution of pericytes across different tissues.

Tissue/Organ	Morphology	Pericyte-to-EC ratio	Distinctive characteristics	Reference
General features	Oval nucleus with longitudinal processes extending along the microvascular axis and spanning several adjacent ECs; distal processes branch and taper to encircle the microvascular wall	—	Located on the abluminal surface of the EC basement membrane; maintain vascular stability via paracrine and physical interactions	(Ayloo et al., 2022; Attwell et al., 2016)
Central nervous system	Flat, stellate	1:1–1:3	Highest vascular coverage; embedded within astrocyte-derived vascular basement membranes	(Drozd et al., 2025; Zheng et al., 2020)
Lung	Fusiform or stellate	1:7–1:9	—	(Wilson et al., 2018)
Heart	Antler-like, with slender filopodia	1:2–1:3	Second most abundant non-cardiomyocyte population after ECs	(Su et al., 2021; Nees et al., 2011)
Liver	Stellate, with long processes wrapping around hepatic sinusoids	1:8–1:10	Liver-resident pericytes located between hepatocytes and sinusoidal ECs; uniquely capable of vitamin A storage	(Li and Fan, 2023; Marcos and Correia-Gomes, 2016; Du et al., 2024)
Kidney	Rounded; circumferentially line capillaries and form close junctions with ECs	≈1:2.5	Include tubular pericytes, podocytes, and mesangial cells; modulate vasomotor tone and vascular permeability	(Smith et al., 2012; Zhang et al., 2020; Humphreys et al., 2010)

### 3.2 Surface marker molecules of renal pericytes

Pericytes exhibit marked heterogeneity, and their morphological and structural characteristics change dynamically in response to tissue-specific localization, developmental stage, and stimuli from the local microenvironment, resulting in substantial differences in surface-marker expression profiles. To date, well-documented characteristic cell-surface markers of pericytes mainly include platelet-derived growth factor receptor β (PDGFR-β/CD140B), neuron-glial antigen 2 (NG2), CD146, and CD13 (Armulik et al., 2011), as well as mesenchymal stem cell-associated markers such as CD90 and CD105 (Guimarães-Camboa et al., 2017); intracellular signature markers include α-smooth muscle actin (α-SMA), non-muscle myosin, desmin, vimentin, and nestin (Zhang et al., 2020).

PDGFRβ is the most widely used marker for identifying renal pericytes. It is stably expressed in renal pericytes under both physiological and pathological conditions (Winkler et al., 2010) and is markedly upregulated upon renal injury and fibrosis (Feng et al., 2025; Chen et al., 2011). However, PDGFRβ is not exclusive to pericytes, and its limitations in the kidney are evident at multiple levels. Glomerular mesangial cells, often regarded as specialized intraglomerular pericytes, also stably express PDGFRβ, yet they differ from renal interstitial pericytes in anatomical localization and function. In mesangioproliferative glomerular diseases, PDGFRβ upregulation largely reflects mesangial cell activation and proliferation rather than changes in interstitial pericytes (Floege et al., 2008). Vascular smooth muscle cells (VSMCs) of afferent arterioles likewise express PDGFRβ and, together with pericytes, constitute the PDGFRβ^+^ mural cell lineage (Andrae et al., 2008). Within the fibrotic microenvironment, the expanded interstitial PDGFRβ^+^ population comprises cells of both pericyte and fibroblast origins (Chen et al., 2011). Even in focal segmental glomerulosclerosis, parietal epithelial cells can inducibly express PDGFRβ (Jia et al., 2023). Single-cell and spatial multi-omic studies of human kidneys have likewise confirmed that PDGFRB is broadly distributed across pericytes, VSMCs, mesangial cells, and multiple fibroblast subpopulations (Abedini et al., 2024; Kuppe et al., 2021; Lake et al., 2023). Therefore, PDGFRβ is more appropriately regarded as a pan-marker of the renal mural or mesenchymal lineage, and renal pericytes should not be identified solely on the basis of PDGFRβ immunostaining or PDGFRβ-Cre lineage tracing.

NG2 is a marker of pericytes during development. It is highly expressed in pericytes of neonatal kidneys but is barely detectable in quiescent pericytes of healthy adult kidneys (Shaw et al., 2018; Stallcup, 2018). Following renal injury, fibrosis, or angiogenesis, pericytes re-express NG2 at high levels (Feng et al., 2025; Kittikulsuth et al., 2021). However, NG2 expression in the kidney is not restricted to pericytes. In glomeruli of healthy adult mice, NG2 is detectable in podocytes, mesangial cells, and parietal epithelial cells (PECs), and its expression in these cell types is further upregulated in a podocyte-depletion model of focal segmental glomerulosclerosis (FSGS) (Suzuki et al., 2018). This indicates that NG2 positivity alone cannot distinguish renal interstitial pericytes from resident glomerular cells. Moreover, a subset of renal NG2^+^ cells exhibits a macrophage-like phenotype and participates in the repair process following ischemic kidney injury (Kittikulsuth et al., 2021), suggesting that NG2^+^ signals in renal tissue may partly originate from infiltrating or resident immune cells rather than pericytes. Taken together, NG2 should be regarded as a dynamic marker of pericyte activation and immaturity rather than a lineage-specific marker of pericytes. Even when the NG2/PDGFRβ double-positive phenotype has been adopted as a signature of activated renal pericytes, confounding by resident glomerular cells and immune cells cannot be fully excluded.

CD146 is a pan-pericyte marker that has been validated in pericytes from multiple tissues; nevertheless, research on and applications of CD146 in renal pericytes remain limited. Studies using embryonic kidney culture models have verified that CD146-positive cells exert targeted regulatory effects on the development of renal ECs (Halt et al., 2016). *In vivo* investigations in mouse kidneys have further shown that CD146-positive pericytes co-localize with NG2 and PDGFRβ and are situated close to vascular structures (Shaw et al., 2021), confirming that CD146 can reliably label renal pericytes in mice. However, CD146 expression in renal tissue is not restricted to pericytes. Under pathological conditions, CD146 has been reported as a marker of endothelial cell activation and injury in patients with chronic renal failure (Malyszko et al., 2004). Notably, in the fibrotic kidneys of CKD patients and unilateral ureteral obstruction (UUO) mice, CD146 is expressed predominantly in renal interstitial vascular endothelial cells; the number of CD146+ endothelial cells correlates positively with the degree of renal interstitial fibrosis, and a subset of these cells is undergoing endothelial-to-mesenchymal transition (EndMT) (Zhang et al., 2024). Therefore, the identification of renal pericytes should rely on combinatorial marker strategies, such as co-localization of CD146, NG2, and PDGFRβ combined with CD31 negativity to exclude endothelial contamination, and, where feasible, be further validated by lineage tracing or single-cell/spatial transcriptomics. Taken together, CD146 may serve as a vascular wall marker. In the fibrotic kidney, CD146^+^CD31^+^ cells should be interpreted as activated or EndMT-committed vascular endothelial cells rather than pericytes.

α-SMA is a widely used marker of the contractile phenotype of pericytes. Pericytes in healthy kidneys exhibit negligible or extremely low α-SMA expression (Kennedy-Lydon et al., 2013). In response to renal injury, pericytes undergo phenotypic transition and differentiate into myofibroblasts, with robust upregulation and intense positivity of α-SMA accompanying this process (Rudman-Melnick et al., 2024). Accordingly, aberrantly elevated α-SMA expression serves as an early key indicator of the phenotypic conversion of pericytes into myofibroblasts. In the normal kidney, VSMCs express high levels of α-SMA, whereas glomerular mesangial cells do not express α-SMA under physiological conditions but can inducibly express it and acquire a contractile phenotype in mesangioproliferative glomerular diseases (Ichimura et al., 2006). α-SMA is essentially a universal functional marker of myofibroblasts, and the origins of renal α-SMA+ myofibroblasts are highly heterogeneous. Lineage-tracing studies have demonstrated that approximately 50% of these cells arise from the proliferation of resident fibroblasts, with the remainder derived from bone marrow-derived cells, endothelial-to-mesenchymal transition, and epithelial-to-mesenchymal transition (LeBleu et al., 2013; Zeisberg et al., 2008). Macrophage-to-myofibroblast transition (MMT) also contributes to the α-SMA+ cell pool (Liang et al., 2022; Tang et al., 2020). Therefore, α-SMA positivity reflects the acquisition of a contractile myofibroblast phenotype but does not indicate a pericyte origin. Taken together, α-SMA should be defined as a functional marker of contractile and activated phenotypes rather than a lineage-specific marker of pericytes.

In recent years, the extensive application of scRNA-seq and spatial transcriptomics in nephrological research has provided technical support for systematically screening highly specific molecular signatures of renal pericytes, thereby overcoming the limitations of traditional markers. Accumulating studies have demonstrated that human renal pericytes are enriched in SLC6A12 and SLC19A1, although the specificity of these markers is lower than that observed in central nervous system pericytes (Sziraki et al., 2023). Screening analysis based on scRNA-seq of mouse pericytes from multiple tissues have confirmed that Myh11 and Kcna5 are expressed with high specificity in mouse renal pericytes (Baek et al., 2022). Integrated scRNA-seq and spatial transcriptomic analysis of human kidneys have further revealed that ITGA8 and POSTN serve as specific markers of glomerular mesangial cells, which are restricted to the glomeruli and are not abundantly expressed in renal interstitial pericytes. By contrast, MYH11, NOTCH3 and NTRK3 are specifically localized to peritubular capillaries, vascular walls and renal interstitial tissues, making them highly specific molecular markers of renal interstitial pericytes (Abedini et al., 2024). **Table 2** summarizes the conventional and newly identified markers used to identify renal pericytes.

**Table 2:** Conventional and newly identified markers of renal pericytes.

Pericyte marker	Comment	Reference
PDGFRβ (CD140b)	The most widely used renal pericyte marker, upregulated upon injury; also expressed by mesangial cells, VSMCs, fibroblasts, parietal epithelial cells	(Abedini et al., 2024; Andrae et al., 2008; Chen et al., 2011; Feng et al., 2025; Floege et al., 2008; Jia et al., 2023; Kuppe et al., 2021; Lake et al., 2023; Winkler et al., 2010)
NG2 (CSPG4)	A developmental marker re-expressed in activated pericytes; also expressed by glomerular resident cells and macrophage-like cells; a dynamic activation marker rather than a lineage-specific marker	(Feng et al., 2025; Shaw et al., 2018; Stallcup, 2018; Suzuki et al., 2018; Kittikulsuth et al., 2021)
CD146 (MCAM)	A pan-pericyte marker co-localizing with NG2 and PDGFRβ in mouse kidneys; in fibrotic kidneys predominantly marks interstitial endothelial cells undergoing EndMT, requiring combinatorial identification with CD31 negativity	(Malyszko et al., 2004; Shaw et al., 2021; Zhang et al., 2024; Halt et al., 2016; Kennedy-Lydon et al., 2013)
CD13 (ANPEP)	A characteristic surface marker, typically used in combination with other markers	(Armulik et al., 2011)
CD90, CD105	Mesenchymal stem cell-associated markers expressed by pericytes with progenitor properties	(Armulik et al., 2011; Guimarães-Camboa et al., 2017)
α-SMA (ACTA2)	A functional marker of the contractile/activated phenotype, upregulated during PMT; a universal myofibroblast marker of heterogeneous cellular origins, not pericyte lineage-specific	(Ichimura et al., 2006; LeBleu et al., 2013; Liang et al., 2022; Rudman-Melnick et al., 2024; Tang et al., 2020; Zeisberg et al., 2008)
Desmin, vimentin, nestin, non-muscle myosin	Intracellular signature markers; none is pericyte-specific individually	(Zhang et al., 2020)
SLC6A12, SLC19A1	Human renal pericyte-enriched markers identified by scRNA-seq; with lower specificity than CNS pericyte markers	(Sziraki et al., 2023)
Myh11, Kcna5	Mouse renal pericyte markers with high organ specificity from multi-tissue scRNA-seq screening	(Baek et al., 2022)
MYH11, NOTCH3, NTRK3	Specific human renal interstitial pericyte markers identified by scRNA-seq and spatial transcriptomics	(Abedini et al., 2024)
ITGA8, POSTN	Specific glomerular mesangial cell markers restricted to the glomeruli; not highly expressed in interstitial pericytes	(Abedini et al., 2024)

In summary, no single marker currently available can specifically define renal pericytes. Conventional markers such as PDGFRβ, NG2, CD146, and α-SMA either co-label other renal cell populations or merely reflect dynamic activation states. Accurate identification of renal pericytes therefore relies on combinatorial marker strategies that integrate multiple conventional markers with novel signature molecules identified by single-cell and spatial transcriptomics (e.g., MYH11, NOTCH3, and NTRK3), ideally complemented by lineage tracing and morphological evidence.

### 3.3 Physiological functions of renal pericytes in renal homeostasis

#### 3.3.1 Structural support and maintenance of vascular morphology

Renal pericytes wrap around ECs through their cytoplasmic processes, providing mechanical support and maintaining the structural integrity of microvascular walls (Dessalles et al., 2021). Slender primary processes extend from the pericyte soma and run parallel to the longitudinal axis of blood vessels, with microtubules forming their principal cytoskeletal component. Secondary processes branch perpendicularly from the primary processes and encircle vessels circumferentially in ring-like or spiral arrangements (Dessalles et al., 2021; Sims, 1986); enriched in actin filaments, these processes constitute the main apparatus for generating contractile force and regulating vascular luminal diameter (Alarcon-Martinez et al., 2018; Peppiatt et al., 2006). Together, the primary and secondary processes form a dual-function cytoskeletal network that supports both structural stability and contractility, thereby preserving microvascular morphology and wall tension. Dense pericyte coverage enhances the ability of microvessels to withstand acute injury. In renal injury models, diphtheria toxin-mediated ablation of PDGFRβ^+^ pericytes induces peritubular capillary endothelial cell death and capillary rarefaction, directly demonstrating the important role of renal pericytes in maintaining endothelial survival and vascular structural integrity (LeBleu et al., 2013).

#### 3.3.2 Regulation of microcirculatory blood flow

Pericyte processes extend around capillaries and cover approximately 90% of the capillary surface area in an organized pattern. Because they are enriched in contractile proteins, pericytes are recognized as major effector cells regulating renal microcirculatory blood flow (Alarcon-Martinez et al., 2020; Beierwaltes et al., 2013). The expression of α-SMA, vimentin and desmin in pericytes facilitates the assembly of actin filament bundles, thereby modulating vasoconstriction and vasodilation (Alarcon-Martinez et al., 2019). *In vitro* studies have confirmed that freshly isolated microvessels exhibit detectable pericyte contraction and changes in luminal diameter within seconds of exposure to vasoactive mediators (Puro, 2007). Renal pericytes have also been identified as renin-producing cells. Because the renin–angiotensin system is the central pathway regulating renal perfusion and systemic blood pressure, this finding further emphasizes the important role of pericytes in maintaining renal microcirculatory homeostasis and controlling blood flow (Stefanska et al., 2016). Co-culture experiments involving renal pericytes and microvascular ECs, together with *in vivo* investigations, have shown that pericytes stabilize the vasculature by secreting angiopoietin-1 (Ang-1) under physiological conditions. Specific genetic ablation of pericytes increases renal vascular permeability and induces renal dysfunction (Lemos et al., 2016). During persistent renal injury, pericytes detach from the vascular wall, causing capillary rarefaction, reduced microcirculatory perfusion, and tissue hypoxia, which ultimately promote the progression of renal fibrosis (Kramann et al., 2017).

#### 3.3.3 Modulation of angiogenesis and vascular maturation

Pericytes function as pioneer cells during angiogenic sprouting and guide endothelial migration. Co-culture of pericytes with ECs and astrocytes promotes the formation of capillary-like structures (Ramsauer et al., 2002). Signaling cascades, including Ang-1/tyrosine-protein kinase receptor TIE 2 (Tie2), PDGF/PDGFR, and vascular endothelial growth factor (VEGF), have been shown to mediate pericyte-dependent regulation of angiogenesis and vascular stabilization. The PDGF-BB/PDGFRβ axis regulates pericyte recruitment (Chiaverina et al., 2019). Proangiogenic interventions such as exogenous VEGF act synergistically with pericyte-endothelial crosstalk to enhance peritubular capillary neovascularization and renal repair (Ding et al., 2025). Pericyte-secreted Ang-1 contributes to vascular stabilization, whereas angiopoietin-2 (Ang-2) acts as an antagonist of Ang-1, inducing vascular destabilization and pericyte detachment (Akwii et al., 2019). These findings indicate that the balance between Ang-1 and Ang-2 is an important regulator of pericyte-mediated vascular maturation. The extent of pericyte coverage also represents an important biomarker of vascular maturation; renal pericytes promote this process by synthesizing basement membrane proteins and secreting growth factors through paracrine mechanisms. Certain subpopulations of renal pericytes possess mesenchymal stem cell (MSC) properties (Kramann and Humphreys, 2014) and can replenish differentiated interstitial and vascular cells lost following injury, suggesting an essential role of pericytes in renal vascular homeostasis and regeneration (Shaw et al., 2018).

#### 3.3.4 Paradigm shift in the understanding of renal pericytes: from structural support cells to hubs of cellular communication

Early studies regarded renal pericytes merely as structural support cells of vascular walls and overlooked their biological functions in actively secreting signaling molecules and remodeling the microenvironment. Through their multi-tiered cytoplasmic processes, renal pericytes simultaneously establish contact with vascular ECs, renal tubular epithelial cells (RTECs) and interstitial fibroblasts, thereby providing an anatomical basis for multicellular signal communication within the kidney. Renal pericytes express and secrete diverse growth factors and exosomal components at high levels, forming an intricate autocrine and paracrine regulatory network. Pericytes secrete chemokine (C-X-C motif) ligand 12 (CXCL12), which activates the C-X-C chemokine receptor type 4/7 (CXCR4/7) signaling cascade in RTECs and promotes tubular epithelial-mesenchymal transition (EMT) (Schreibing and Kramann, 2022; Tang et al., 2019a). This pericyte-tubular epithelial crosstalk permits long-distance regulation of nephron function. Bidirectional secretion of interleukin-10 (IL-10) and transforming growth factor-beta (TGF-β) by renal pericytes regulates macrophage polarization. Meanwhile, pericytes release proinflammatory mediators, including monocyte chemoattractant protein-1 (MCP-1) and interleukin-6 (IL-6), to recruit macrophages and other immune cells (Leaf et al., 2017), thereby amplifying local inflammatory responses and promoting pericyte-to-myofibroblast transition (PMT). Accordingly, communication between pericytes and immune cells acts as a critical molecular switch regulating renal inflammatory activation and fibrotic progression. In addition, renal pericytes deliver nucleic acids, proteins, and lipid mediators through exosomes and microvesicles, enabling long-distance intercellular and even interorgan endocrine signaling (Shi et al., 2020). Collectively, these lines of evidence demonstrate that renal pericytes are not merely structural accessory cells but critical signaling hubs that coordinate homeostasis of the renal microenvironment.

## 4 Phenotypic transition of pericytes in renal fibrosis

During renal fibrosis, pericytes lose their homeostatic phenotype, detach from the vascular wall, migrate, become activated, and transition into a myofibroblast-like phenotype, while also contributing to capillary rarefaction and interstitial remodeling. This phenotypic transition not only provides a source of myofibroblasts but also represents a critical pathological link between microvascular injury and fibrotic progression.

### 4.1 Pericyte detachment and migration

Pericytes detach from the endothelial surface, lose tight junctions, and migrate into the renal interstitium. This positional shift marks their transition from maintainers of vascular homeostasis to reactive interstitial cells and constitutes an initiating event in renal fibrosis. Transcriptomic analysis has revealed significant downregulation of genes associated with cell junctions and anchoring junctions in injured pericytes, supporting the loss of endothelial contact (Kramann and Humphreys, 2014). Lineage-tracing studies have demonstrated that, in the UUO model, pericyte processes become shorter, the area of contact with ECs decreases, and pericytes gradually migrate toward interstitial regions (Humphreys et al., 2010). After detachment, pericytes acquire migratory capacity and enhanced responsiveness, allowing them to react to local injury and inflammatory signals.

### 4.2 Pericyte-to-myofibroblast transition

Under sustained injury, numerous pericytes become activated, upregulate α-SMA, and transition into myofibroblast precursors; these cells can be designated myofibroblast progenitor cells. The origin of myofibroblasts has remained a long-standing question in renal fibrosis research. Early studies proposed that epithelial cells directly acquire a myofibroblast-like phenotype through EMT; however, subsequent lineage-tracing investigations have provided complementary evidence. In UUO and ischemia-reperfusion injury (IRI) models, pericyte-specific LacZ labeling has revealed that interstitial α-SMA^+^ cells originate from pericytes (Humphreys et al., 2010). Multicolor labeling has further demonstrated that PDGFRβ^+^/α-SMA^–^ pericytes are the predominant source of myofibroblasts in tubulointerstitial fibrosis in the UUO model and gradually convert into α-SMA^+^ myofibroblasts during disease progression (He et al., 2025; Buhl et al., 2020). Although current techniques cannot label every precursor population, pericytes undoubtedly constitute a major source of myofibroblasts. Renal pericytes can stabilize capillary networks *in vitro*, whereas myofibroblasts derived from fibrotic kidneys lose this capacity (Schrimpf et al., 2012), indicating attenuation of the vascular support function of pericytes and enhancement of their contractile, matrix-depositing, and interstitial-remodeling capacities. Following detachment from the vascular wall, pericytes not only serve as a source of myofibroblasts but also promote capillary instability and rarefaction. The resulting reduction in capillary density aggravates local hypoxia, oxidative stress, and inflammation, which further promote pericyte activation, interstitial cell expansion, and ECM deposition, establishing a vicious cycle of “vascular loss–hypoxia–fibrosis”. Therefore, PMT contributes to the development of peritubular capillary rarefaction and the progression of renal fibrosis.

## 5 Renal pericyte-mediated cellular interactions

### 5.1 Pericyte-endothelial cell interactions

Under normal conditions, pericytes envelop the outer surface of capillaries, share a basement membrane with ECs, and maintain structural and functional coupling. Through direct contact and local support, pericytes maintain ECs in a state of low activation, thereby limiting increases in vascular permeability (Lemos et al., 2016) and inflammatory cell adhesion (Tanaka et al., 2023). This supportive effect is not unidirectional but represents a dynamic balance through which pericytes and ECs jointly maintain vascular wall homeostasis (Lin et al., 2011).

#### 5.1.1 Microvascular destabilization induced by pericyte detachment

Under mild or reversible injury, pericytes and ECs can jointly participate in adaptive vascular remodeling rather than dissociating immediately. Pericytes regulate EC survival, local perfusion, and capillary remodeling, enabling the microvascular network to mount compensatory responses to hypoxia, inflammation, and metabolic stress (Jourde-Chiche et al., 2019; Shaw et al., 2018). During persistent renal injury, the coordinated interaction between pericytes and ECs gradually deteriorates. Endothelial dysfunction induces pericyte detachment, proliferation, and migration (Perry and Okusa, 2016). Following kidney injury, pericytes rapidly upregulate the metalloproteinase ADAMTS1 while downregulating its endogenous inhibitor TIMP3, thereby driving pericyte detachment from peritubular capillaries and their migration into the interstitium. In three-dimensional culture, renal pericytes bind to and stabilize capillary tube networks and suppress metalloproteolytic activity in endothelial cells, whereas myofibroblasts derived from these pericytes lose this vessel-stabilizing function (Schrimpf et al., 2012). Together, these findings provide direct evidence that pericyte detachment from the vessel wall contributes to microvascular instability in the kidney. Various cytokines within the inflammatory microenvironment can also directly disrupt pericyte-endothelial junctions, thereby aggravating detachment. Tumor necrosis factor-α (TNF-α)-activated RTECs release Indian Hedgehog ligand, which disrupts pericyte-vessel connections, promotes microvascular rarefaction, and drives local fibrosis (O'Sullivan et al., 2023). Thus, disruption of pericyte-endothelial crosstalk is closely associated with renal microvascular rarefaction.

#### 5.1.2 Interaction imbalance drives the transition from vascular injury to fibrosis

Dysregulated crosstalk between pericytes and ECs serves as a critical pivot in the transition from renal microvascular injury to fibrosis. Upon detachment of pericytes from the walls of peritubular capillaries, ECs lose the structural stabilization and anti-proteolytic protection conferred by pericytes, and the microvasculature progressively undergoes rarefaction; concurrently, the detached pericytes transdifferentiate into myofibroblasts, thereby directly contributing to renal interstitial fibrosis. Following injury, hyperactivation of PDGFRβ and VEGFR2 results in pericyte detachment, microvascular instability, leakage, and rarefaction; importantly, blockade of either signal through soluble receptors simultaneously ameliorates capillary rarefaction and renal fibrosis (Lin et al., 2011). Mechanistically, PMT is accompanied by a switch in VEGF isoform secretion—from the pro-angiogenic VEGF164 toward the dys-angiogenic isoforms VEGF120 and VEGF188—providing direct evidence that altered pericyte behavior can trigger EC loss (Lin et al., 2011). Hypoxia-inducible factor (HIF) serves as a major transcription factor in renal pericytes responding to hypoxic stress and regulates angiogenesis and vascular remodeling by upregulating vascular regulatory factors such as VEGF and Ang1 (Troise et al., 2024). Marked HIF accumulation upregulates the expression of TGF-β1 and Notch3 in ECs, thereby inducing glomerular arteriolar remodeling accompanied by aberrant pericyte recruitment and increased mural coverage of the vessel wall, which in turn promotes PMT and renal interstitial fibrosis (Wang et al., 2017b). This evidence further indicates that dysregulated pericyte–endothelial crosstalk can manifest as “excessive coverage” (e.g., arterialization-like remodeling) rather than microvascular rarefaction alone. **Figure 2** summarizes the mechanisms through which pericyte**–**endothelial cell interactions regulate renal fibrosis.

**Figure 2:**
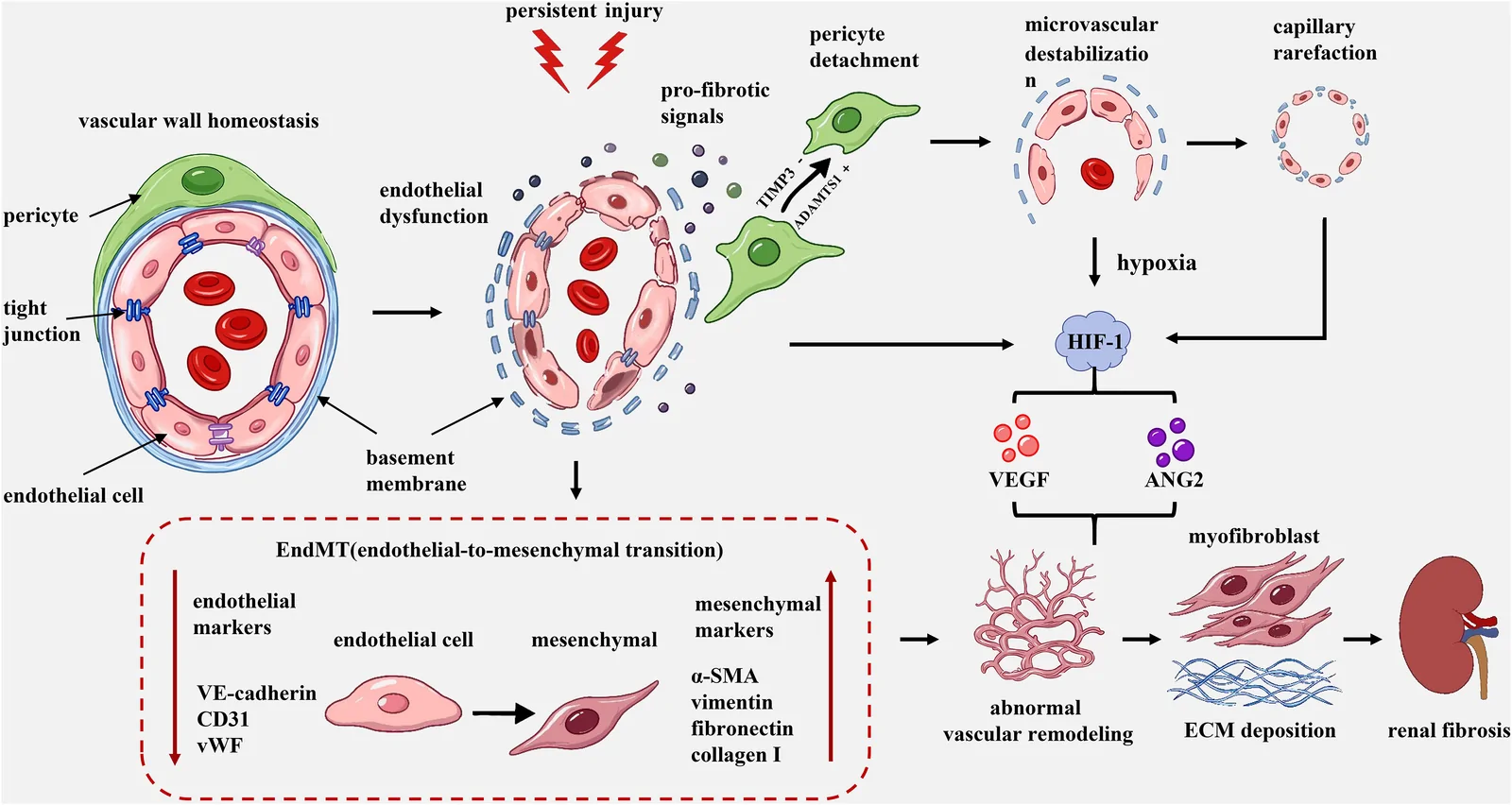
Pericyte**–**endothelial cell interactions in the regulation of renal fibrosis. Renal injury disrupts pericyte**–**endothelial coupling and induces pericyte detachment, microvascular rarefaction, and PMT, which collectively establish a self-perpetuating hypoxia**–**fibrosis cycle. Solid black arrows, interactions with direct renal evidence; dashed black arrows, interactions with indirect renal evidence; dashed green arrows, interactions supported by cross-organ evidence from non-renal models.

### 5.2 Pericyte-renal tubular epithelial cell interactions

In the normal kidney, the structural coupling between pericytes and RTECs is less intimate than that between pericytes and ECs; nevertheless, dynamic homeostasis is maintained through paracrine signaling, bidirectional signal exchange across the basement membrane, and alterations in the vascular microenvironment. When RTECs undergo persistent injury, this balance becomes disrupted. RTECs then become a predominant source of pathological signals, while pericytes shift from a homeostatic state to an activated state, ultimately amplifying focal epithelial damage into widespread interstitial fibrosis.

#### 5.2.1 Tubular epithelial cell injury drives pericyte activation and phenotypic switching

Injury to RTECs serves as a primary trigger for pericyte activation and phenotypic switching. In Coll-GFP transgenic mice subjected to UUO, nuclear localization of phosphorylated Smad2 was observed concurrently in RTECs and Coll-GFP^+^ pericytes (Wu et al., 2013). Paracrine induction of Smad2 phosphorylation in pericytes by TGF-β1 released from injured RTECs provides direct evidence that RTECs injury drives pericyte activation. Studies using Hedgehog reporter mice have demonstrated that the ligands Shh and Ihh are expressed in RTECs, whereas the effector molecules glioma-associated oncogene homolog 1/2 (Gli1/2) are expressed exclusively in adjacent PDGFRβ^+^ interstitial pericytes and fibroblasts, establishing a well-defined paracrine signaling circuit. In renal fibrosis models, Ihh is upregulated, Gli1^+^ interstitial cells undergo an 11-fold expansion, and subsequently differentiate into α-SMA^+^ myofibroblasts (Fabian et al., 2012); this process is abrogated by RTEC-specific deletion of Ihh (O'Sullivan et al., 2023). Proximal tubule-derived wingless/integrase-1 (Wnt1) drives interstitial fibrosis independently of inflammation, and activation of Wnt signaling in interstitial pericytes likewise promotes myofibroblast activation (Maarouf et al., 2016). Collectively, these studies establish that injured RTECs act on neighboring PDGFRβ^+^ pericytes through three principal paracrine axes—TGF-β1, Hedgehog ligands (Shh/Ihh), and Wnt ligands—driving their proliferation and PMT, thereby fueling a vicious cycle of capillary rarefaction and interstitial fibrosis.

#### 5.2.2 Activated pericytes reciprocally regulate tubular epithelial injury and aberrant repair

Upon activation, pericytes detach from peritubular capillaries (PTCs), leading to PTC destabilization, degeneration, and rarefaction (Schrimpf et al., 2012); the resulting microvascular loss is thought to drive sustained interstitial hypoxia and, secondarily, oxidative stress, ATP depletion, and apoptosis in RTECs (Fine and Norman, 2008). Following PMT, pericytes become the predominant source of interstitial myofibroblasts and produce large quantities of ECM components, such as collagen I/III and fibronectin. This process leads to interstitial fibrosis and thickening of the tubular basement membrane, indirectly inducing RTEC atrophy, apoptosis, and luminal obliteration, thereby disrupting the normal architecture and function of the renal tubules (Fligny and Duffield, 2013). Activated pericytes further secrete profibrotic factors, including TGF-β and Wnt, which induce partial EMT and cellular senescence in RTECs (Luo et al., 2018; DiRocco et al., 2013), thereby aggravating tubular epithelial injury and amplifying fibrotic signaling. **Figure 3** summarizes the mechanisms through which pericyte**–**renal tubular epithelial cell interactions regulate renal fibrosis.

**Figure 3:**
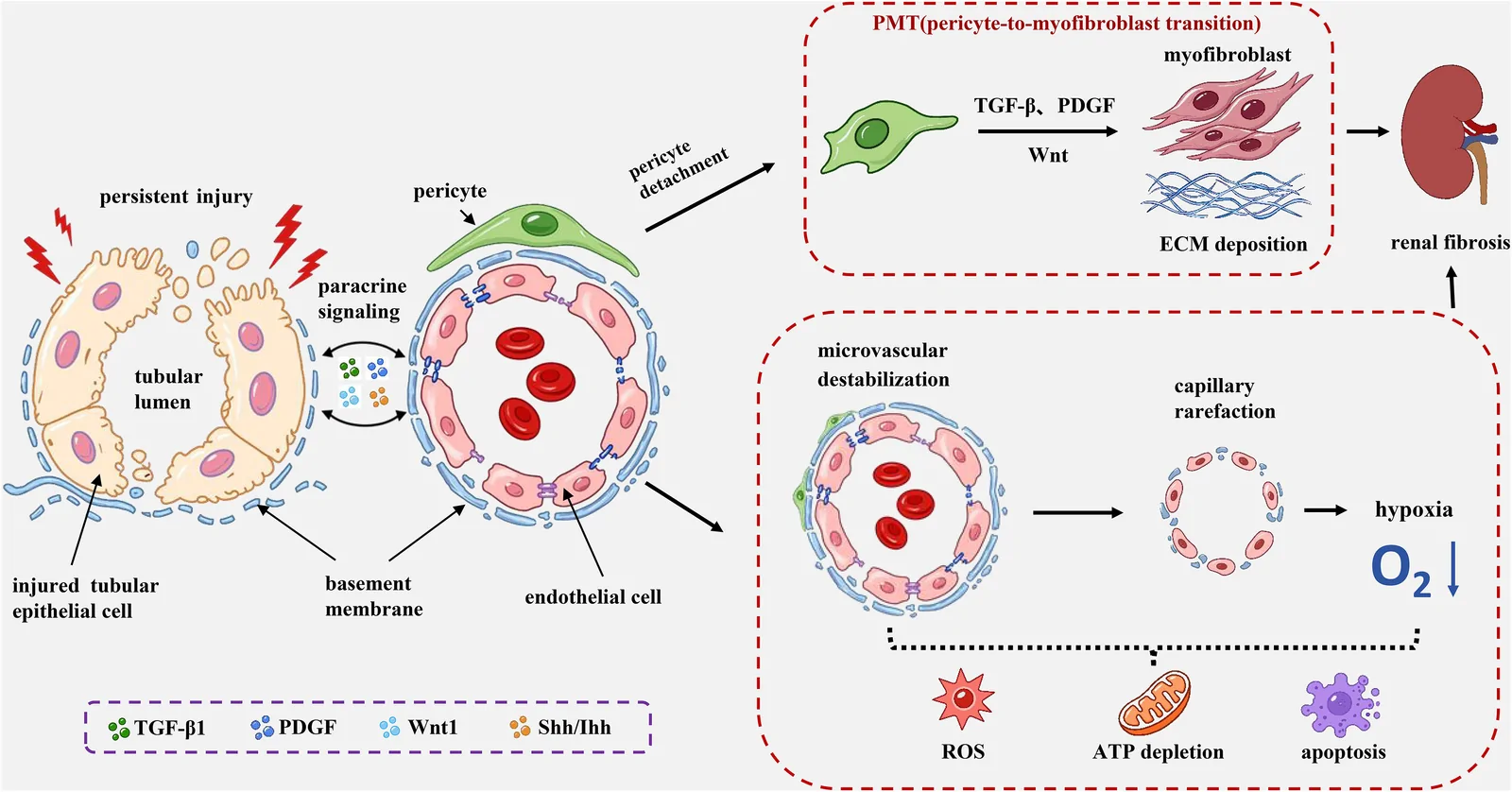
Pericyte–renal tubular epithelial cell interactions in the regulation of renal fibrosis. Injured RTECs activate pericytes through paracrine TGF-β1, CTGF, and Hedgehog signaling; activated pericytes, in turn, aggravate tubular hypoxia, EMT, and ECM deposition, forming a bidirectional profibrotic loop. Solid black arrows, interactions with direct renal evidence; dashed black arrows, interactions with indirect renal evidence; dashed green arrows, interactions supported by cross-organ evidence from non-renal models.

### 5.3 Pericyte-immune cell interactions

Interactions between pericytes and immune cells are centered on the injury response and become markedly enhanced under pathological conditions such as renal ischemia, inflammation, toxic injury, and aberrant tissue repair. Following kidney injury, pericytes regulate immune cell recruitment and infiltration by remodeling local microenvironmental homeostasis and the inflammatory mediator network. Infiltrating immune cells, particularly monocytes/macrophages, reciprocally regulate pericyte activation, proliferation, and migration and induce their transition toward a profibrotic phenotype. These reciprocal regulatory processes constitute a bidirectional pathogenic crosstalk mechanism.

#### 5.3.1 Pericyte involvement in inflammatory cell recruitment

MCP-1 is a key chemokine secreted by pericytes and exerts potent chemotactic effects on immune cells expressing its receptor, C-C motif chemokine receptor 2 (CCR2) (Shibahara et al., 2020). Following kidney injury, MCP-1 is abundantly produced by RTECs, ECs, and activated renal pericytes (Chatterjee et al., 2024), thereby recruiting circulating monocytes to the injury sites. In addition, pericytes secrete macrophage inflammatory proteins (MIP-1α/CCL3 and MIP-1β/CCL4) and CXCL chemokines (e.g., IL-8/CXCL8, CXCL9, and CXCL10), which synergistically recruit neutrophils, T cells, and additional macrophages, collectively establishing a complex inflammatory microenvironment (Kaushik et al., 2021). Renal pericytes also secrete classical proinflammatory cytokines, including IL-6, TNF-α, and interleukin-1β (IL-1β), which regulate immune cell activation and differentiation, activate endothelial cells, promote leukocyte adhesion and extravasation, and stimulate ECM deposition (Tanaka, 2024; Leaf et al., 2017).

#### 5.3.2 Expression of immunoregulatory receptors by pericytes

Renal pericytes intrinsically express various immunoregulatory receptors, enabling them to receive and respond to complex signals originating from immune cells and the microenvironment and thereby participate in bidirectional and multidirectional intercellular communication. PDGFRβ is not only the most canonical marker of pericytes, but also a critical regulatory receptor in these cells; hyperactivation of the PDGFRβ signaling pathway constitutes an important mechanism driving PMT. Furthermore, PDGFRβ contributes to the regulation of the immunomodulatory functions of pericytes, as PDGF-BB stimulation markedly increases MCP-1 production by pericytes, thereby enhancing their ability to recruit and activate macrophages, which in turn aggravates inflammation and fibrosis (Chatterjee et al., 2024; Moon et al., 2025). Renal pericytes also express toll-like receptor 4 (TLR4), which recognizes lipopolysaccharide (LPS) and various endogenous damage-associated molecular patterns, including high-mobility group box 1 (HMGB1) and heat shock proteins (HSPs) (Vallés et al., 2023). In sepsis-induced acute kidney injury (AKI), elevated circulating levels of LPS-binding protein have been shown to activate TLR4 signaling in renal pericytes, representing a key initiating event for PMT induction (Castellano et al., 2019). Additionally, renal pericytes express complement receptors such as C5a receptor 1 (C5aR1) (Sahu et al., 2022). Following complement activation, the anaphylatoxin C5a binds to C5aR1 on pericytes, directly promoting their transition into myofibroblasts and enhancing their production of inflammatory mediators (Peng et al., 2019). Collectively, these functionally diverse receptors enable renal pericytes to integrate multiple injury- and immune-derived signals, positioning them as critical participants in the fibrotic process.

#### 5.3.3 Pericyte-macrophage interactions

Bidirectional communication between pericytes and macrophages begins during the early stage of kidney injury and persists throughout renal fibrosis. Pericytes represent a substantial source of MCP-1 within the kidney and effectively capture and recruit CCR2^+^ monocytes/macrophages (Chatterjee et al., 2024; Leaf et al., 2017). Several lines of kidney-specific direct evidence support pericyte-macrophage crosstalk in renal fibrosis. In models of UUO, expression of the the NLR family pyrin domain containing 3 (NLRP3) inflammasome and IL-1β following renal injury was shown to be predominantly confined to kidney pericytes, which drive inflammatory cell infiltration through chemokine secretion. Pericyte-specific ablation of Pericyte-specific ablation of myeloid differentiation primary response protein 88 (MyD88)—which disrupts the TLR2/4-MyD88/IRAK4 inflammatory program—markedly attenuated renal injury and fibrosis (Leaf et al., 2017), indicating that pericyte-intrinsic signaling promotes macrophage recruitment. In parallel, PDGF-BB stimulation strongly upregulated CCL2 (MCP-1) and colony-stimulating factor 1 (CSF1) in renal pericytes, two factors essential for monocyte chemotaxis and survival, respectively; pharmacological blockade of PDGFRβ signaling with imatinib reduced renal interstitial macrophage infiltration and fibrosis (Chen et al., 2011), further supporting that activated renal pericytes recruit macrophages via CCL2/CSF1 secretion.

The polarization status of macrophages profoundly affects the function and fate of pericytes. During the early phase of kidney injury, signals such as LPS and interferon-gamma (IFN-γ) within the local microenvironment induce recruited monocytes to differentiate into classically activated M1 macrophages. These cells act as major inflammatory effectors, releasing abundant proinflammatory cytokines and partially activating pericytes, thereby making them more susceptible to subsequent profibrotic signals (Leng et al., 2024; Jiang et al., 2024). Although M2 macrophages possess anti-inflammatory and tissue-repair functions, they predominantly display profibrotic properties during renal fibrosis (Wang et al., 2021). TGF-β1 and PDGF secreted by M2 macrophages bind to TGF-β receptors and PDGFRβ on pericytes, enabling the pericytes to synthesize and secrete large amounts of collagen and other ECM components and promoting their proliferation and differentiation into myofibroblasts (Al-Orjani et al., 2025; Long et al., 2023). Collectively, these findings indicate that macrophages not only indirectly regulate PMT through paracrine pathways but also directly function as effector cells in renal fibrosis through their own phenotypic conversion.

#### 5.3.4 Pericyte-T lymphocyte interactions

Different T-cell subsets exert bidirectional regulatory effects on renal fibrosis by secreting specific cytokines. T helper 17 (Th17) cells are proinflammatory effector cells whose sustained activation following AKI is associated with progressive fibrosis (Mehrotra et al., 2015); in contrast, regulatory T cells (Tregs) exert immunosuppressive functions, and their accumulation attenuates renal injury and promotes tissue repair (Lee et al., 2024). A spatial multi-omic single-cell atlas of the human kidney identified 51 distinct cell types and 28 disease-associated cell states in healthy and diseased renal tissues, including CD4+ T cells, CD8+ T cells, vascular smooth muscle cells, and pericyte populations, providing direct evidence for the coexistence of T lymphocytes and pericytes in the kidney (Lake et al., 2023). Furthermore, a single-cell atlas of human kidney fibrosis delineated the pericyte-lineage origin of myofibroblasts and their spatial niche relationships with immune cells, revealing the spatial proximity of these cell types and thereby providing a structural basis for paracrine interactions between T lymphocytes and pericytes (Kuppe et al., 2021). Nevertheless, the specific functional interactions between these two cell populations remain insufficiently characterized. TGF-β1 is a key cytokine linking adaptive immunity to tissue fibrosis, acting on mesenchymal cells to drive profibrotic transcriptional programs (Budi et al., 2021). In the kidney, TGF-β1 induces the PMT. Although experimental evidence that T cell–derived TGF-β1 acts directly on renal pericytes is currently lacking, pericytes express TGF-β receptors and are responsive to TGF-β1; they could therefore, in principle, also respond to T cell–derived TGF-β1, an inference that awaits further experimental validation. Evidence from non-renal fields demonstrates that Rgs5 deficiency normalizes pericyte coverage of tumor vessels, thereby promoting T cell infiltration into tumor tissue and immune-mediated clearance (Hamzah et al., 2008), indicating that pericytes regulate T cell tissue infiltration and that their coverage status constitutes a regulatory node for extravascular T cell migration. This provides a hypothetical basis for inferring that alterations in the microenvironment may modulate renal pericyte coverage and thereby regulate interstitial T cell infiltration.

#### 5.3.5 Pericyte–neutrophil interactions

Neutrophils are among the first immune cells recruited to sites of injury in AKI. The temporal pattern of chemokine expression following renal ischemia-reperfusion parallels leukocyte infiltration and the progression of tissue damage and repair, being most pronounced during the reparative phase (Stroo et al., 2010). In the UUO model, a rapidly expanding Siglec-F^+^ neutrophil subset in the injured kidney directly expresses type I collagen, serving as a substantial immune-derived source of collagen in the fibrotic kidney and contributing to ECM accumulation (Zheng et al., 2025; Ryu et al., 2022). UUO induces gasdermin D (GSDMD) activation in neutrophils, resulting in extensive production of neutrophil extracellular traps (NETs), which induce MMT through the GSDMD/TGF-β1/Smad signaling pathway and participate in and amplify renal interstitial fibrosis. The above evidence directly demonstrates the involvement of neutrophil subsets in renal fibrosis. Research on how pericyte-neutrophil interactions regulate renal fibrosis remains limited, whereas studies in other organs have revealed modes of interaction between these two cell types. Direct analysis of leukocyte-pericyte interactions in inflamed tissues by confocal intravital microscopy has shown that, following transendothelial migration, neutrophils crawl along pericyte processes and reach the gaps between adjacent pericytes in an intercellular adhesion molecule 1 (ICAM-1)-, Mac-1-, and LFA-1-dependent manner; under inflammatory conditions, pericyte shape changes enlarge these gaps, which then serve as exit points for neutrophils breaching the venular wall (Proebstl et al., 2012), providing evidence that pericytes directly regulate neutrophil extravascular migration. Given that peritubular capillaries are likewise composed of endothelial cells and pericytes, gaps formed by pericyte-endothelial dissociation during acute kidney injury may similarly serve as channels for neutrophil infiltration. Although current evidence has not directly demonstrated interactions between renal pericytes and neutrophil subsets or NETs, given the pivotal role of pericytes in neutrophil recruitment, it is plausible that pericytes establish a neutrophil-rich microenvironment that favors NET formation and its subsequent profibrotic pathological effects. Nevertheless, direct evidence is still lacking as to whether renal pericytes are direct target cells of NETs and whether NETs drive the PMT, which warrants further experimental investigation.

#### 5.3.6 Pericyte–mast cell interactions

The renal interstitium exhibits substantial enrichment of the mast cell marker tryptase beta 2 (TPSB2) (Barwinska et al., 2021), indicating that mast cells constitute a resident cell population within this compartment. Mast cell degranulation releases mediators, including histamine, tryptase, and chymase, that disrupt the renal microvascular barrier and promote inflammatory extravasation (Martins et al., 2025; Wernersson and Pejler, 2014). Mast cell deficiency attenuates UUO-induced renal fibrosis, an effect that can be reversed by mast cell reconstitution (Summers et al., 2012) providing direct evidence that mast cells promote renal fibrosis. However, research on how pericyte–mast cell interactions regulate renal fibrosis in the kidney remains limited. Studies in other organs have nevertheless revealed complex and important modes of interaction between these two cell types. Mast cell-derived mediators, including histamine, serotonin, VEGF, IL-6, and IL-8, act synergistically to induce contraction of pericytes and ECs, resulting in the formation of intercellular gaps (Majno et al., 1969; Kelley et al., 1988). In models of allergic asthma, tryptase released upon mast cell activation induces the retraction of pulmonary microvascular pericytes, loss of adhesion molecules, and loss of pericyte coverage, providing direct cross-organ evidence that mast cell proteases affect pericyte cytoskeleton and contractility (Joulia et al., 2024). Given that the microvasculature of both the lung and kidney is composed of endothelial cell–pericyte units, the mechanism by which mast cell proteases disrupt pericyte coverage may be hypothetically extrapolated to peritubular capillary rarefaction and pericyte detachment in the kidney. **Figure 4** summarizes the mechanisms through which pericyte**–**immune cell interactions regulate renal fibrosis.

**Figure 4:**
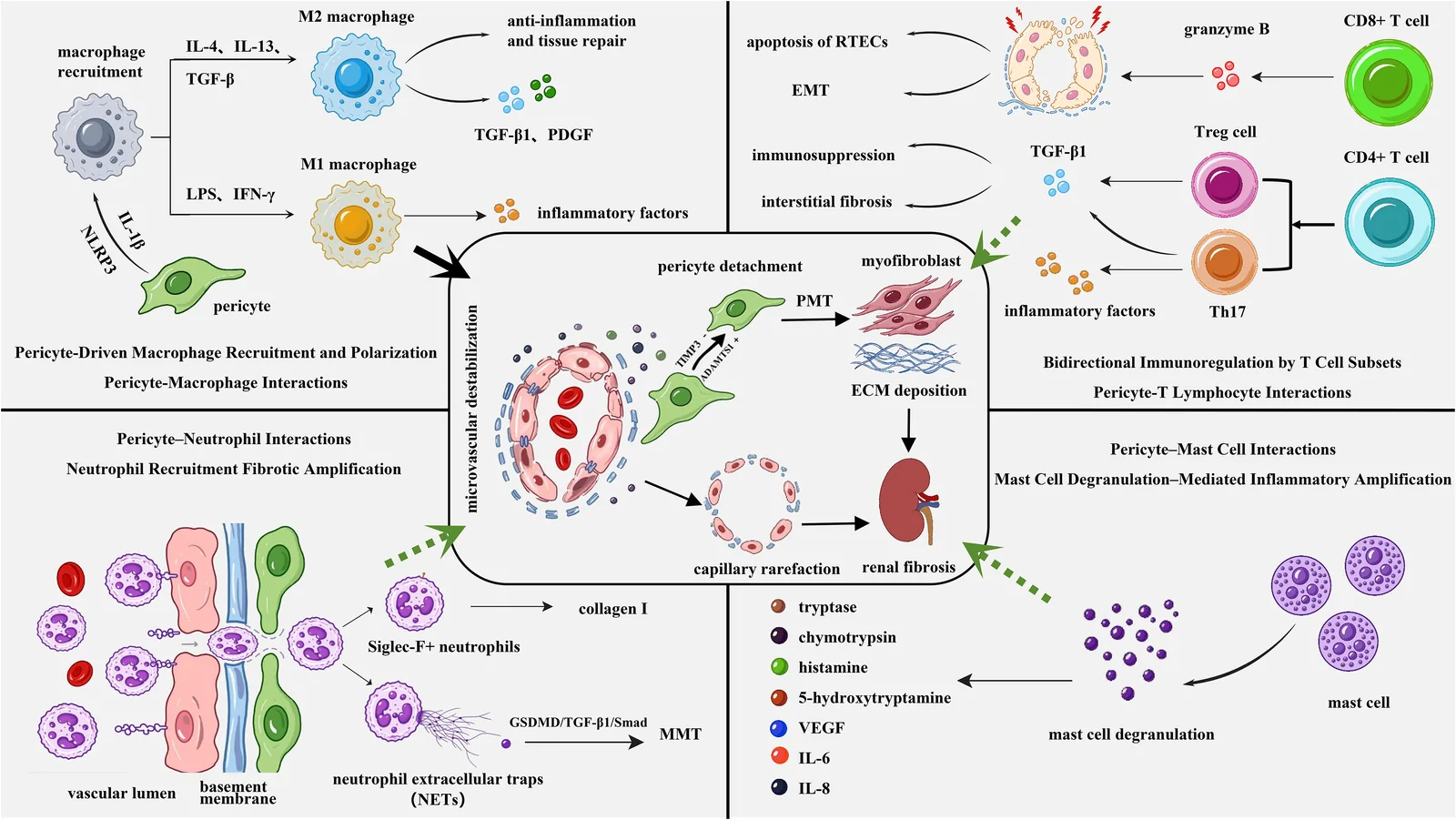
Pericyte**–**immune cell interactions in the regulation of renal fibrosis. Pericytes recruit macrophages, neutrophils, T cells, and mast cells through chemokines and cytokines, whereas immune cell**-**derived mediators reciprocally promote pericyte activation, PMT, and fibrotic amplification. Solid black arrows, interactions with direct renal evidence; dashed black arrows, interactions with indirect renal evidence; dashed green arrows, interactions supported by cross-organ evidence from non-renal models.

### 5.4 Pericyte–fibroblast interactions

Pericytes and fibroblasts are the two major mesenchymal cell populations within the renal interstitium. Following renal injury, both cell types can transition into myofibroblasts and synergistically drive the progression of renal fibrosis through paracrine signaling, direct cell–cell contact, and ECM-mediated interactions.

#### 5.4.1 Lineage relationship between renal pericytes and fibroblasts

Pericytes and fibroblasts exhibit substantial overlap in their developmental origins, anatomical localization, and functions and can undergo mutual conversion under specific conditions. Forkhead box D1 (FOXD1)-lineage cells in the kidney serve as common progenitors of pericytes, fibroblasts, and myofibroblasts, indicating a close lineage relationship among these cell types (Lathan, 2024). Pericytes reside within the capillary basement membrane, whereas fibroblasts are distributed throughout the interstitium; both belong to the perivascular mesenchymal cell population and are located around peritubular capillaries. Genetic lineage tracing has demonstrated that injury activates FOXD1-lineage pericytes and induces their transition into myofibroblasts (Gomez and Duffield, 2014). scRNA-seq has identified multiple intermediate myofibroblast states, including OGN3^+^COL14A1^+^ and Thy1^+^Adamts1^+^ populations, in fibrotic kidneys from both humans and mice, suggesting that the transition from quiescent fibroblasts to terminally differentiated myofibroblasts is a multistage process (Schreibing et al., 2023). In the resting state, pericytes and fibroblasts retain functional heterogeneity. Following injury, they converge along a shared differentiation trajectory and convert into myofibroblasts, together constituting the major source of ECM deposition during the progression of renal fibrosis.

#### 5.4.2 Paracrine signaling mediates pericyte–fibroblast communication

The interplay between pericytes and fibroblasts is mediated predominantly through paracrine signaling: these two mesenchymal cell populations secrete TGF-β1, connective tissue growth factor (CTGF), PDGF family ligands, and a range of inflammatory mediators, thereby establishing a local regulatory network that jointly drives the progression of renal fibrosis. Single-cell atlases of human kidney fibrosis have resolved the multiple origins of myofibroblasts—including pericytes and fibroblasts—and ligand–receptor analysis suggest the existence of an interaction network involving mediators such as TGF-β1 among mesenchymal cell subpopulations (Kuppe et al., 2021), indirectly supporting a potential bidirectional TGF-β1 loop between fibroblasts and pericytes. Renal fibroblasts have been shown to produce CTGF and IGF-I under high-glucose conditions, and the two factors cooperate to induce collagen production (Lam et al., 2003). CTGF induces renal fibrosis via epidermal growth factor receptor (EGFR) activation and can also potentiate the profibrotic effects of TGF-β1 (Rayego-Mateos et al., 2018), thereby indirectly promoting the phenotypic transition of both pericytes and fibroblasts and amplifying the renal fibrotic response.

In addition to soluble factors, extracellular vesicle (EV)-mediated molecular transfer may represent a novel route of communication between pericytes and fibroblasts. Bone marrow mesenchymal stem cell (BMSC)-derived exosomes carrying miR-34c-5p preferentially accumulate in the injured kidney and enter pericytes and fibroblasts via the CD81-EGFR ligand-receptor complex (Hu et al., 2022b), thereby attenuating renal interstitial fibrosis—demonstrating that both mesenchymal cell types possess EGFR-mediated EV uptake capacity. Although direct reports of natural EV-mediated communication between pericytes and fibroblasts in the kidney remain scarce, studies in non-renal models—such as the penile tunica albuginea model of Peyronie’s disease—have demonstrated that pericyte-derived EV-mimetic nanovesicles can modulate the gene expression profiles of primary cultured fibroblasts (Yin et al., 2021). Tunica albuginea fibrosis and renal interstitial fibrosis are both stromal fibrotic disorders characterized by pericyte activation and excessive ECM deposition. Given that pericyte-derived EVs were shown to directly reshape fibroblast transcriptional programs, a comparable communication mechanism may likewise operate in the kidney. **Figure 5** summarizes the mechanisms through which pericyte**–**fibroblast interactions regulate renal fibrosis.

**Figure 5:**
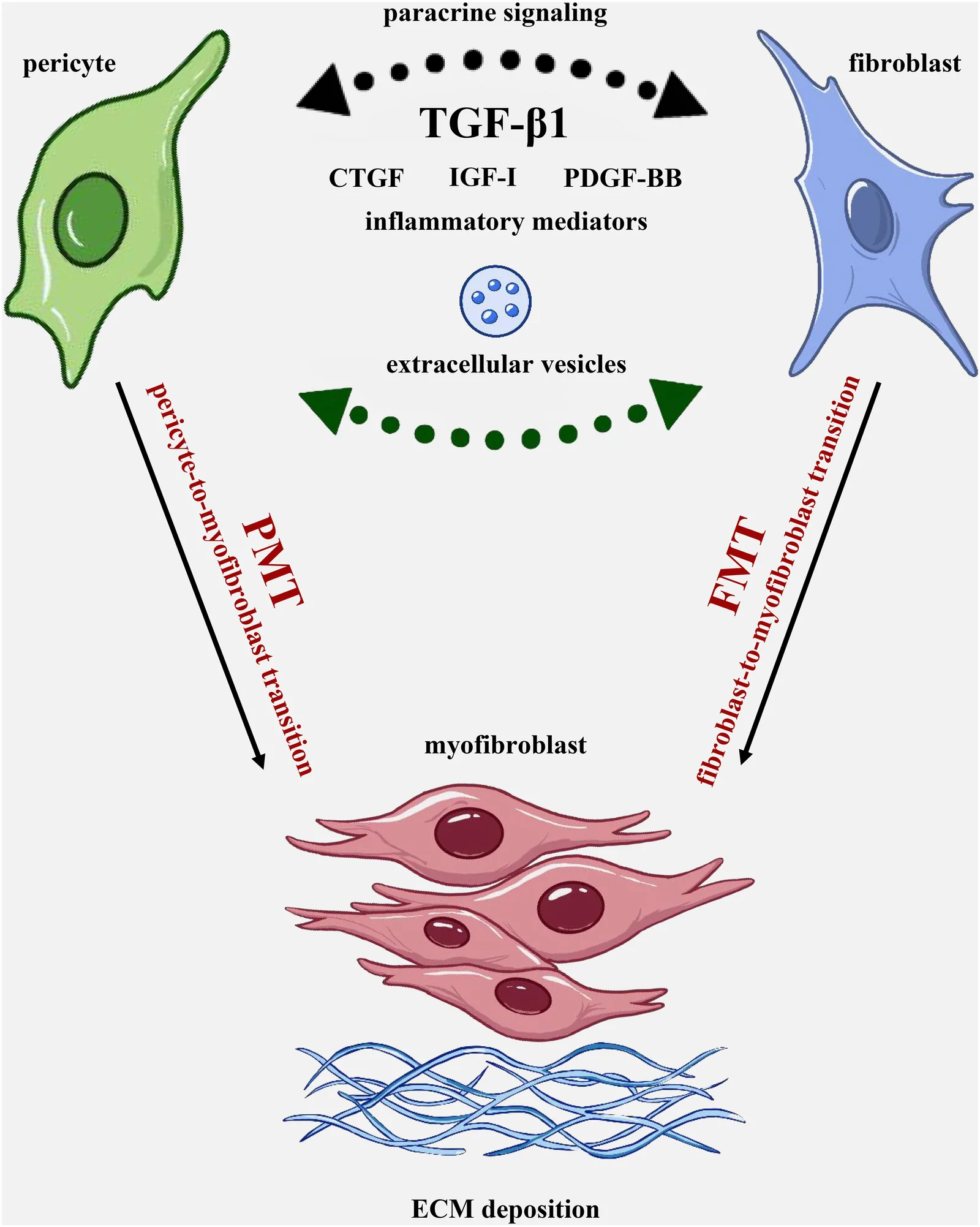
Pericyte**–**fibroblast interactions in the regulation of renal fibrosis. Pericytes and fibroblasts mutually activate each other through paracrine TGF-β1, CTGF, and PDGF-BB signaling, thereby establishing a self-amplifying profibrotic loop. Solid black arrows, interactions with direct renal evidence; dashed black arrows, interactions with indirect renal evidence; dashed green arrows, interactions supported by cross-organ evidence from non-renal models.

## 6 Signaling pathways regulating pericyte-mediated intercellular interactions

### 6.1 Ang/Tie2 signaling pathway

The Ang/Tie2 signaling pathway is a key mechanism governing vascular development, stability, and permeability, comprising the ligands Ang-1 and Ang-2 and their shared receptor Tie2 (Saharinen et al., 2017; Augustin et al., 2009). Notably, Tie2 is specifically expressed in ECs rather than pericytes, indicating that this pathway does not function as a pericyte-intrinsic signaling cascade. Ang-1 is a glycoprotein predominantly produced by renal pericytes and acts on Tie2 receptors expressed by ECs, inducing receptor phosphorylation (Saharinen et al., 2017) and activating downstream cascades such as phosphatidylinositol 3-kinase (PI3K)/protein kinase B (Akt), RhoA/rho-associated coiled-coil-containing protein kinase (ROCK), and nuclear factor kappa B (NF-κB), which collectively stabilize blood vessels and reduce vascular permeability. Under pathological conditions associated with renal injury, endothelial cells release large amounts of Ang-2, resulting in a marked increase in the Ang-2/Ang-1 ratio. Ang-2 competitively inhibits the binding of Ang-1 to Tie2 (Saharinen et al., 2017; Chang et al., 2022), thereby attenuating or abolishing Tie2 signaling, disrupting stable adhesion between pericytes and ECs, and ultimately causing pericyte detachment. Therefore, the Ang/Tie2 signaling pathway is fundamentally an intercellular communication axis between pericytes and endothelial cells. In patients with CKD, plasma Ang-2 levels and the Ang-2/Ang-1 ratio are significantly and positively correlated with declining renal function and progression to kidney failure (David et al., 2010; Chang et al., 2022). In a IRI rat model, Ang-2/Ang-1 imbalance is accompanied by pericyte proliferation and fibrosis (Khairoun et al., 2013), with markedly upregulated Ang-2 expression in local renal tissue, and inhibition of Ang-2 significantly attenuates pericyte–endothelial cell dissociation. Beyond the kidney, anti-Ang-2–specific antibodies reduce retinal pericyte loss in diabetic macular edema and have completed clinical translation (Wykoff et al., 2022; Koller et al., 2026), providing a feasibility reference for the renal field. Taken together, pericytes serve as the Ang-1 ligand–producing cells and downstream effector cells of the Ang/Tie2 axis, through which Ang/Tie2 signaling maintains pericyte–endothelial cell interactions and preserves renal microvascular homeostasis.

### 6.2 PDGF/PDGFRβ signaling pathway

The PDGF family comprises five dimeric isoforms—PDGF-AA, -AB, -BB, -CC, and -DD—which initiate downstream signaling cascades by binding to the tyrosine kinase receptors PDGFRα and PDGFRβ (Fredriksson et al., 2004; Andrae et al., 2008).The ligand PDGF-BB is secreted by multiple cell types, including platelets, endothelial cells, macrophages, fibroblasts, and RTECs (Lv et al., 2018), while the receptor PDGFRβ is likewise expressed in mesangial cells, vascular smooth muscle cells, and fibroblasts (Tanaka et al., 2023). Thus, although this pathway is not pericyte-specific, pericytes constitute one of the key effector cell populations of the PDGF/PDGFRβ axis. PDGFRβ signaling directly drives PMT in the kidney: in isolated renal pericytes, PDGF-BB directly activates PDGFRβ and its downstream extracellular signal-regulated kinases 1 and 2 (ERK1/2) and PI3K/Akt signaling, inducing pericyte proliferation and migration and the acquisition of an α-SMA-positive myofibroblast phenotype (Chen et al., 2011); in models of UUO and IRI, pharmacological blockade of PDGFR signaling inhibits PMT and attenuates renal interstitial fibrosis. The same study further demonstrated that PDGF-BB-activated pericytes upregulate CCL2 and CSF1, thereby promoting monocyte-to-macrophage differentiation and initiating local macrophage infiltration in the kidney, indicating that renal pericytes drive macrophage recruitment (Chen et al., 2011). Moreover, in PDGFRβ-DTR mice permitting selective depletion of renal pericytes, exogenous PDGF-BB upregulated PDGFRβ expression and exacerbated pericyte–endothelial detachment, whereas selective pericyte depletion abolished the protective effect of bone marrow-derived putative endothelial progenitor cells against IR-induced renal injury, further confirming that pericytes serve as effector cells of PDGF-BB (Wang et al., 2020). Sustained PDGFRβ signaling weakens the close association between pericytes and endothelial cells and induces pericyte contraction and detachment from the vascular basement membrane, thereby amplifying inflammatory and fibrotic signals and ultimately culminating in irreversible renal fibrosis.

### 6.3 TGF-β/Smad signaling pathway

The TGF-β/Smad signaling axis is a key pathway governing renal fibrosis: upon binding to the type I/II TGF-β receptors (TβRI/TβRII), TGF-β1 induces Smad2/3 phosphorylation, which forms a complex with Smad4 and translocates into the nucleus to drive the transcription of profibrotic target genes, including α-SMA and type I collagen (Derynck and Zhang, 2003). In parallel, TGF-β1 can activate non-Smad pathways, including ERK1/2, p38, and c-Jun N-terminal kinase (JNK), via adaptor proteins such as TNF receptor-associated factor 6 (TRAF6) and Src homology and collagen A (ShcA) (Derynck and Budi, 2019). Because TβRI/TβRII are broadly expressed in tubular epithelial cells, endothelial cells, macrophages, mesangial cells, and fibroblasts, TGF-β/Smad is not a pericyte-specific pathway. Lineage-tracing studies have established pericytes/perivascular mesenchymal cells as the predominant source of myofibroblasts in the fibrotic kidney (Kramann et al., 2015; Humphreys et al., 2010); thus, pericytes represent one of the key effector cells of this pathway, although pathway activation per se can be achieved through multiple routes. In the UUO model, TGF-β1 is produced predominantly by injured RTECs, while downstream receptor signaling is elevated in both epithelial cells and pericytes; the TβRI inhibitor SB431542 markedly blocks renal PMT, exemplifying paracrine epithelial–pericyte crosstalk (Wu et al., 2013). In diabetic kidney disease, reduced pericyte expression of integrin β8 leads to increased TGF-β1 release and activation, driving pericyte transition via the pericyte-intrinsic TGF-β1/TGFBR1/Smad3 pathway, which directly demonstrates that TGF-β/Smad executes profibrotic effects within pericytes (Cao et al., 2024). TGF-β signaling can also influence pericyte behavior indirectly: endothelial-specific blockade of TGF-β signaling reduces EndMT, thereby maintaining renal pericyte coverage and microvascular stability (Xavier et al., 2015); and TGF-β1/Smad3 signaling drives M2 macrophage polarization, with macrophages serving as a major source of TGF-β1 in the fibrotic kidney whose paracrine action on pericytes may further promote PMT (Wang et al., 2016). Within renal pericytes, TGF-β/Smad and PDGF/PDGFRβ signaling are functionally complementary: TGF-β1 predominates in driving PMT, whereas PDGF provides the mitogenic signal for pericyte proliferation and detachment from the vascular wall. PDGF/PDGFRβ signaling promotes pericyte detachment and activates downstream ERK1/2, thereby forming a positive feedback loop with the TGF-β pathway (Wang et al., 2017a).

### 6.4 Hippo-YAP/TAZ signaling pathway

The core kinase cascade of the Hippo-Yes-associated protein (YAP)/transcriptional co-activator with PDZ-binding motif (TAZ) pathway consists of mammalian STE20-like kinases 1/2 (MST1/2) and large tumor suppressor 1/2 (LATS1/2): LATS1/2 phosphorylates YAP/TAZ, thereby retaining it in the cytoplasm and targeting it for degradation; upon pathway inactivation or cellular sensing of mechanical stress, YAP/TAZ is dephosphorylated and translocates into the nucleus, where it binds TEAD transcription factors to activate target genes such as CTGF (Ccn2), CYR61 (Ccn1), and α-SMA (Acta2) (Fu et al., 2022) YAP/TAZ is a ubiquitously expressed mechanosensitive transcriptional coactivator that can be activated in RTECs, ECs, fibroblasts, and pericytes alike. Specific deletion of Yap/Taz in the perivascular mesenchymal lineage using Gli1-CreER markedly attenuated UUO-induced ECM deposition and myofibroblast accumulation, providing direct evidence that YAP/TAZ exerts its profibrotic effects through renal pericytes. Notably, nuclear translocation of YAP/TAZ precedes α-SMA upregulation following injury, suggesting that it constitutes an early triggering event in the PMT (Liang et al., 2017). Beyond promoting PMT, YAP/TAZ activation also modulates ECs function, thereby indirectly regulating pericyte behavior. Endothelial-specific YAP activation promotes EndMT, which secondarily disrupts pericyte–endothelial attachment, whereas endothelial-specific YAP deletion significantly attenuates UUO-induced EndMT and renal fibrosis (Ren et al., 2021). Furthermore, YAP/TAZ regulates Ang-2 expression, vascular sprouting, and vascular barrier maturation; endothelial YAP/TAZ deficiency leads to elevated Ang-2 levels and impaired pericyte coverage (Kim et al., 2017). Concurrently, YAP/TAZ activation promotes MMP production by pericytes, thereby degrading basement membrane components and disrupting endothelial-pericyte adhesion (Kim et al., 2022), which further aggravates vascular permeability. In addition to crosstalk with the Ang/Tie2 pathway that indirectly maintains microvascular homeostasis, the Hippo-YAP/TAZ pathway also exhibits synergistic effects with the TGF-β/Smad signaling pathway. In addition, TGF-β1 can activate the noncanonical RhoA/ROCK pathway, which inhibits LATS1/2 and promotes YAP/TAZ dephosphorylation, thus regulating renal PMT (Szeto et al., 2016; Miranda et al., 2021).

### 6.5 Wnt/β-Catenin signaling pathway

Wnt ligands are a family of lipid-modified, secreted glycoproteins; upon binding to Frizzled receptors and LRP5/6 co-receptors, they inhibit the β-catenin destruction complex composed of APC, Axin, GSK-3β, and CK1, thereby stabilizing β-catenin and allowing its nuclear translocation, where it associates with T-cell factor (TCF)/lymphoid enhancer factor (LEF) transcription factors to activate target gene transcription (He et al., 2009; Edeling et al., 2016). During renal fibrosis, multiple Wnt ligands—including Wnt1, Wnt3a, Wnt4, Wnt5a, Wnt7b, and Wnt10b—are markedly upregulated (He et al., 2009), originating from injured tubular epithelial cells, interstitial cells, and myeloid immune cells (Tian et al., 2024; Edeling et al., 2016). Although Frizzled/LRP receptors are broadly expressed across resident renal cell types and Wnt/β-catenin is therefore not a pericyte-specific pathway, pericytes constitute one of its key effector cells. Conditional activation of canonical Wnt/β-catenin signaling in interstitial pericytes using the FOXD1 lineage tool is sufficient to induce spontaneous myofibroblast differentiation and renal fibrosis in the absence of injury (DiRocco et al., 2013), directly demonstrating that β-catenin drives PMT within pericytes. Beyond this direct effect, Wnt ligand upregulation in renal fibrosis can also indirectly affect pericyte interactions with other cells. In obstructive renal fibrosis, upregulation of multiple Wnt ligands and nuclear β-catenin accumulation in interstitial cells promote basement membrane degradation and disrupt pericyte–endothelial cell adhesion (Lovisa et al., 2020). In addition, Wnt/β-catenin signaling promotes macrophage M2 polarization and proliferation (Feng et al., 2018), amplifying inflammation and thereby facilitating renal PMT.

### 6.6 Notch signaling pathway

The Notch signaling pathway is a cell–cell contact-dependent signal transduction system comprising four Notch receptors (Notch1-4) and five canonical ligands (Delta-like 1, 3, 4 and Jagged 1, 2). Upon ligand–receptor binding, the receptor is cleaved by γ-secretase to release the Notch intracellular domain (NICD), which translocates into the nucleus and forms a transcriptional complex with RBPJ and MAML to activate target genes such as Hes/Hey (Siebel and Lendahl, 2017). In the kidney, Notch signaling influences pericyte behavior predominantly through indirect mechanisms: in renal injury models, endothelial sirtuin 1 (SIRT1) inactivation upregulates delta-like protein 4 (Dll4) and activates Notch signaling, thereby aggravating peritubular capillary rarefaction and fibrosis (Kida et al., 2016). *In vitro* evidence further demonstrates that co-stimulation with Dll4 and TGF-β1 promotes the transdifferentiation of primary renal pericytes into myofibroblasts more potently than TGF-β1 alone (Kida et al., 2016), suggesting a synergistic interplay between Notch and TGF-β/Smad signaling at the pericyte level. In contrast, direct evidence for Notch signaling targeting renal pericytes remains limited. Vascular biology studies have shown that endothelial-derived Jagged1 activates Notch3 in mural cells, establishing an autoregulatory loop that maintains pericyte phenotype and vascular stability (Liu et al., 2009). In idiopathic pulmonary fibrosis (IPF), Notch1 promotes the PMT through the PDGFR/ROCK1 signaling axis (Wang et al., 2019), indicating crosstalk between Notch and PDGF/PDGFRβ signaling that amplifies PMT. Given that fibrosis in both the lung and kidney relies on pericyte-to-myofibroblast conversion and that both organs share PDGFRβ as a pericyte marker and effector node, these mechanisms may provide valuable insights into the Notch-mediated regulation of renal pericytes. **Figure 6** summarizes the signaling pathways that regulate pericyte-mediated intercellular interactions.

**Figure 6:**
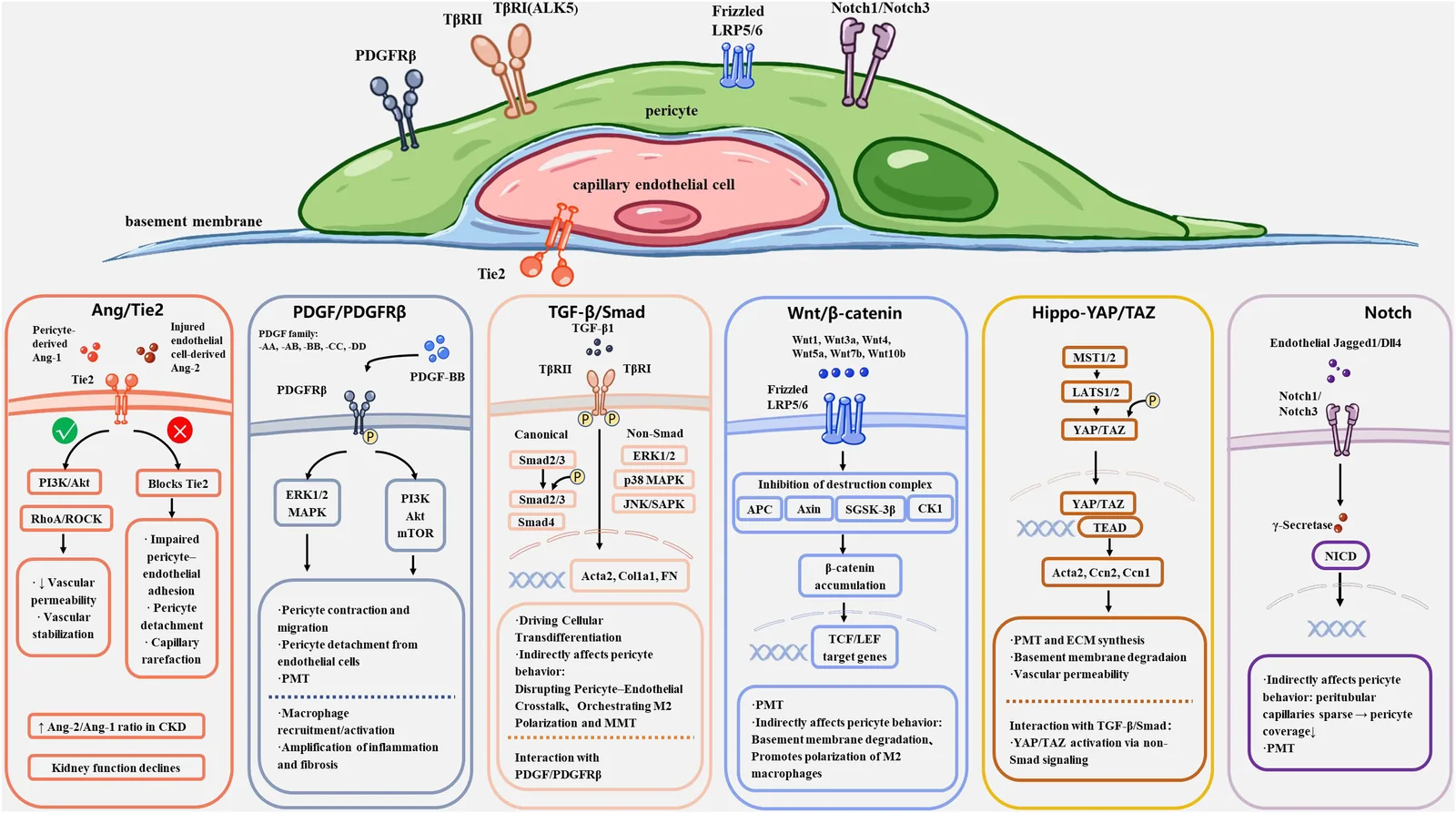
Molecular pathways regulating pericyte-mediated intercellular interactions. The Ang/Tie2, PDGF/PDGFRβ, TGF-β/Smad, Hippo-YAP/TAZ, Wnt/β-catenin, and Notch pathways coordinately regulate pericyte-mediated crosstalk and converge on PMT, EndMT, ECM deposition, and capillary rarefaction. Solid black arrows, interactions with direct renal evidence; dashed black arrows, interactions with indirect renal evidence; dashed green arrows, interactions supported by cross-organ evidence from non-renal models.

## 7 Intervention strategies targeting pericyte interactions

### 7.1 Mesenchymal stem cell therapy

MSCs possess immunomodulatory, pro-angiogenic, and anti-fibrotic properties, and their renoprotective effects are mediated predominantly through paracrine mechanisms. According to the directness of the intervention, the available evidence can be classified into two categories. The first comprises therapies that directly restore pericyte–endothelial cell interactions. In a rat model of IR-induced AKI-to-fibrosis transition, BMSC transplantation preserved PTC density, inhibited pericyte detachment from the endothelium, migration, and transdifferentiation into myofibroblasts, and BMSCs were observed to differentiate directly into pericyte-like cells participating in PTC repair, indicating a dual role in stabilizing pericyte–endothelial interactions and replenishing the pericyte pool (Hu et al., 2022a). In the same model system, BMSC transplantation was further shown to reduce the number of α-SMA^+^ myofibroblasts in the renal interstitium, maintain the normal perivascular distribution of PDGFRβ^+^ pericytes, and, by correcting the imbalance of VEGF isoforms and Ang-1/Ang-2 secreted by injured pericytes, block PMT, a critical node of disrupted intercellular crosstalk (Wang et al., 2023). Studies in organs other than the kidney provide parallel evidence; for instance, intravenous infusion of human umbilical cord-derived MSC-like cells preserved cerebral PDGFRβ^+^ pericyte density and pericyte–endothelial colocalization, thereby maintaining blood–brain barrier integrity (Barretto et al., 2021). Nevertheless, given the structural differences in microvasculature among organs, such cross-organ extrapolations require validation in renal models. The second category encompasses therapies that indirectly modulate pericyte behavior. Since endothelial integrity and PTC survival are prerequisites for pericyte–endothelial homeostasis, adipose-derived MSCs may indirectly stabilize pericytes by secreting VEGF and other factors that promote endothelial proliferation and tube formation (Rautiainen et al., 2021). Hypoxia-preconditioned MSCs, through enhanced VEGF and HGF secretion, markedly attenuated renal fibrosis and inflammatory infiltration while preserving proximal tubular integrity and pericyte coverage (Ishiuchi et al., 2020). Kidney-derived MSC microparticles localized to PTCs in mice with UUO and protected the microvascular bed by inhibiting EndoMT, thereby reducing the stimuli driving pericyte detachment and transdifferentiation (Choi et al., 2015). Regarding translational application, clinical trials of MSCs and MSC-derived EVs have preliminarily confirmed their safety in patients with diabetic kidney disease and CKD (Perico et al., 2026; Nassar et al., 2016); however, these trials have primarily adopted renal function and safety as endpoints, and the pericyte-modulatory mechanism has not yet been directly verified in humans through renal biopsy or biomarkers. In addition, the short *in vivo* survival and low renal homing efficiency of MSCs, together with the lack of batch-to-batch consistency and standardized dosing regimens for vesicle-based formulations, remain to be addressed.

### 7.2 Engineered extracellular vesicles

EVs are bilayer-membrane nanoparticles secreted by cells that mediate intercellular communication by transferring proteins, lipids, and nucleic acids. Native EVs—particularly those derived from MSCs—have demonstrated therapeutic potential in various models of renal fibrosis (Shi et al., 2020; Kholia et al., 2020). However, insufficient targeting specificity and suboptimal renal accumulation limit their clinical application. Engineered EVs (EEVs), bioengineered to enhance targeting, cargo-loading capacity, and therapeutic efficacy, have therefore emerged as a research frontier. With respect to pericytes as a target, EEV studies directly targeting renal pericytes remain scarce, and most available evidence reflects indirect modulation of pericyte behavior. For example, arginyl-glycyl-aspartic acid peptide-modified MSC-EVs (RGD-MSC-EVs), by binding to integrin receptors highly expressed on activated myofibroblasts and pericytes, achieve markedly enhanced accumulation in fibrotic kidneys and, through delivery of miR-126-5p to restore RNA m6A methylation homeostasis, attenuate PTC loss and renal interstitial fibrosis (Zhang et al., 2025). Nevertheless, since their therapeutic mechanism centers on the endothelium–PTC axis and pericytes were not examined separately, the restoration of pericyte–endothelial interactions remains indirect. Similarly, EVs secreted by glial cell line-derived neurotrophic factor (GDNF)-modified human adipose mesenchymal stem cells (hAMSCs) activate endothelial SIRT1/eNOS signaling and reduce PTC loss in tubulointerstitial fibrosis, thereby indirectly preserving pericyte coverage (Chen et al., 2020). Studies in organs other than the kidney provide *in vivo* evidence for direct EEV targeting of pericyte-like cells: pPB peptide-modified umbilical cord MSC-derived exosomes specifically target activated HSCs—the pericyte-like cells of the liver—and attenuate liver fibrosis (Wang et al., 2026); likewise, aminoethyl anisamide (AEAA)-modified exosomes loaded within a hydrogel target activated HSCs via sigma receptor mediation and markedly inhibit hepatic fibrosis (Sun et al., 2025). Collectively, this cross-organ evidence demonstrates the feasibility of EEV strategies directed at activated pericyte-like cells and offers valuable lessons for developing renal pericyte-targeted EEVs. Regarding translational application, the ligand modification, cargo loading, and physical navigation strategies of EEVs directly address the targeting and accumulation bottlenecks of native EVs and thus hold high translational relevance. However, current evidence remains largely at the preclinical small-animal stage, with a lack of large-animal studies and clinical trials in kidney disease; moreover, no pericyte-specific targeting ligands are available, cargo-loading efficiency and quality-control standards have not been established, and surface modifications may introduce immunogenicity risks—all of which remain to be resolved.

### 7.3 Nanomedicine delivery systems

Conventional systemic administration often fails to achieve effective drug concentrations at sites of renal fibrosis; by recognizing specific receptors on renal cells and molecular markers of the fibrotic microenvironment, nanodrug delivery systems offer new avenues for the precise intervention of renal fibrosis (Tang et al., 2019b; Zhang et al., 2026). However, studies directly targeting renal pericytes remain rare, and most available evidence reflects indirect modulation of pericyte behavior. Regarding direct targeting in the kidney, RGD peptide-modified liposomes achieve targeted delivery by exploiting integrin αvβ3, which is highly expressed on the surface of renal interstitial myofibroblasts, and significantly attenuate renal interstitial fibrosis in mice with UUO (Zhou et al., 2021); given that PMT is a major source of myofibroblasts in renal fibrosis, this strategy in essence acts directly on the transdifferentiated progeny of pericytes, thereby blocking pericyte-mediated profibrotic effects. Regarding indirect modulation, platelet membrane-biomimetic platelet membrane-biomimetic poly(lactic-co-glycolic acid) (PLGA) nanoparticles (PMVs@PLGA) loaded with TGF-β1 siRNA exploit the intrinsic inflammation-tropic properties of platelet membranes to achieve passive targeting and accumulation in injured kidneys; by blocking TGF-β1/Smad3 signaling, they inhibit the transmission of profibrotic signals from renal interstitial cells to pericytes while attenuating the autocrine TGF-β1 loop within pericytes (Fei et al., 2024). Beyond the kidney, HSCs, the pericyte-like cells of the liver, have provided direct parallel evidence for nanotargeting: PDGFRβ-binding peptide (pPB)-modified stable nucleic acid lipid nanoparticles can deliver siRNA to activated HSCs (Jia et al., 2018), and PDGFRβ-targeting peptide-modified chitosan nanoparticles can likewise efficiently deliver anti-TGF-β1 siRNA to activated HSCs via PDGFRβ-mediated endocytosis, effectively suppressing liver fibrosis (Mostafa et al., 2025). Although these strategies have so far been validated only in models of liver fibrosis, their targeting mechanisms offer a potentially translatable new paradigm for the prevention and treatment of renal fibrosis. In terms of translational application, nanomedicine is a relatively mature delivery platform, yet direct evidence for pericyte targeting in the kidney is limited to isolated studies, and no targeted nanodrug for renal fibrosis has entered clinical investigation to date (Zhang et al., 2026). The main limitations are as follows: existing targeting ligands are not pericyte-specific and cannot distinguish pericytes from other activated interstitial cells; an inherent contradiction exists between renal targeting efficiency and glomerular filtration clearance, with stringent cutoffs imposed on particle size and charge; and the delivery efficiency and off-target effects of siRNA cargoes await systematic evaluation. Moreover, current renal studies lack pericyte-specific efficacy readouts such as pericyte coverage and PMT, making it difficult to distinguish direct effects from indirect benefits.

### 7.4 Natural product–based therapies

Natural products, characterized by multi-target and multi-pathway actions, low toxicity, and holistic regulation, exhibit unique advantages against renal fibrosis. The available evidence can be classified into therapies that directly target PMT and those that indirectly modulate pericyte behavior. As direct evidence, Shenkang injection—a modern preparation of Chinese patent medicine containing anthraquinones such as emodin and aloe-emodin—dose-dependently attenuated tubulointerstitial fibrosis in a rat model of obstructive nephropathy, with its mechanism explicitly directed at PMT and its signaling activation, thereby providing direct evidence that compound herbal formulations can counteract renal fibrosis by inhibiting PMT (Liu et al., 2019). As indirect evidence, injured RTECs release profibrotic factors such as TGF-β1 through EMT, constituting the upstream trigger that drives PMT in neighboring pericytes: rhubarb extract attenuated renal fibrosis in adenine-induced CKD rats by inhibiting the TGF-β/Smad pathway and ameliorating metabolomic disturbances (Zhang et al., 2018); artesunate alleviated renal fibrosis by restoring expression of the anti-aging protein Klotho and suppressing TGF-β1/Smad signaling (Mohammad et al., 2025); the combination of asiatic acid and naringenin additively inhibited renal fibrosis in obstructive nephropathy by rebalancing Smad3/Smad7 (Meng et al., 2015); and salvianolic acid C attenuated renal tubulointerstitial fibrosis in the UUO model by suppressing Smad3 and the EMT transcription factor Snail (Wu et al., 2023). All of these therapies indirectly limit TGF-β1-dependent PMT by interrupting the transmission of RTEC-derived profibrotic signals. In addition, coptisine alleviated inflammation, oxidative stress, and mitochondrial apoptosis in renal tubular epithelium via PI3K/Akt signaling (Liu et al., 2022), and berberine ameliorated renal inflammation and fibrosis by suppressing NLRP3 inflammasome activation and EMT (Ma et al., 2022); given that NLRP3 inflammasome activation is an important inflammatory signal driving PMT, suppressing inflammation and oxidative stress may likewise indirectly attenuate inflammation-driven pericyte activation. As cross-organ evidence, the Astragalus–Salvia herb pair promoted pericyte recruitment in mice with myocardial ischemia and maintained vascular homeostasis by activating the Ang-1/Tie2/focal adhesion kinase (FAK) axis, thereby promoting angiogenesis and vessel maturation in the ischemic region and directly regulating pericyte–endothelial interactions (Zhang et al., 2022); given that renal fibrosis and myocardial ischemia share microvascular rarefaction and Ang-1 downregulation, this finding offers important implications for restoring pericyte–endothelial interactions in the kidney. Regarding translational application, Shenkang injection, as a marketed Chinese medicinal preparation, has been widely used in patients with CKD, and agents such as artesunate and berberine are already in clinical use; nevertheless, several limitations remain: the composition of herbal formulae is complex, with active ingredients and dose–effect relationships poorly defined; some natural compounds exhibit low oral bioavailability and unclear renal exposure, and natural products are not invariably safe; mechanistic studies are heavily concentrated on the TGF-β/Smad axis; and most natural monomers are supported only by animal experiments, with no randomized controlled clinical trials targeting renal fibrosis conducted to date.

## 8 Conclusion

In summary, renal pericytes are not only essential for maintaining microvascular structural and functional homeostasis but also, owing to their unique anatomical location and secretory capacity, serve as critical communication hubs linking ECs, RTECs, immune cells, and interstitial fibroblasts. Pericyte detachment from the vascular wall, together with their activation and PMT, constitutes an important pathological link between microvascular rarefaction and progressive interstitial fibrosis. The signaling cascades reviewed herein—including PDGF/PDGFRβ, TGF-β/Smad, Ang/Tie2, Wnt/β-catenin, Hippo-YAP/TAZ, and Notch—collectively establish a pericyte-centered intercellular communication network, and emerging interventions such as MSC therapy, EEVs, targeted nanocarriers, and natural products offer promising avenues for modulating this network.

Nevertheless, several limitations of the current evidence should be acknowledged. First, no single marker currently permits the specific identification of renal pericytes; commonly used markers such as PDGFRβ are also expressed by resident fibroblasts, and lineage-tracing techniques are unable to label all precursor populations, which complicates the precise delineation of pericyte fate and subpopulation heterogeneity. Second, most mechanistic evidence is derived from rodent models such as UUO and IRI. Given the interspecies differences between rodents and humans in pericyte biology, disease progression patterns, and therapeutic responses, direct extrapolation of these findings to human CKD remains limited. Third, several intercellular interactions—such as pericyte communication with T lymphocytes and mast cells, as well as EV-mediated pericyte–fibroblast communication—are largely inferred from spatial proximity or extrapolated from non-renal models, and direct functional validation in the kidney is still lacking. Fourth, therapeutic strategies targeting pericyte-mediated intercellular crosstalk remain predominantly at the preclinical stage. Iissues such as targeting specificity, renal accumulation efficiency, dosing regimens, long-term safety, and scalable manufacturing remain unresolved, and evidence from human clinical trials is still absent.

Future research may proceed in several directions. First, priority should be given to defining highly specific marker combinations, integrated with single-cell and spatial multi-omics analysis of human kidney tissues, to resolve pericyte heterogeneity and validate findings from animal studies in clinical specimens. Second, intercellular interactions that remain insufficiently characterized should be subjected to direct functional validation using approaches such as co-culture systems, kidney organoids, and vesicle tracing, with particular attention to the dynamic changes and stage-specific roles of pericyte subpopulations in order to identify optimal therapeutic windows. At the translational level, efforts should focus on developing pericyte-targeted delivery vehicles, standardized and scalable manufacturing of MSC- and engineered EV-based products, and rationally designed combination regimens, thereby advancing toward rigorously designed clinical trials. Collectively, these efforts will be essential for developing pericyte-centered antifibrotic strategies with clinical applicability.

## Glossary

CKD: chronic kidney disease
ESRD: end-stage renal disease
ECM: extracellular matrix
scRNA-seq: Single-cell RNA sequencing
PRISMA: Preferred Reporting Items for Systematic Reviews and Meta-Analyses
EC: endothelial cell
HSCs: hepatic stellate cells
PDGFR-β: platelet-derived growth factor receptor-beta
PDGF: platelet-derived growth factor
NG2: neuron-glial antigen 2
α-SMA: α-smooth muscle actin
VSMCs: vascular smooth muscle cells
UUO: unilateral ureteral obstruction
EndMT: endothelial-to-mesenchymal transition
MMT: macrophage-to-myofibroblast transition
Ang: angiopoietin
Tie2: tyrosine-protein kinase receptor TIE 2
VEGF: vascular endothelial growth factor
MSC: mesenchymal stem cell
RTECs: renal tubular epithelial cells
CXCL: chemokine (C-X-C motif) ligand
CXCR4/7: C-X-C chemokine receptor type 4/7
EMT: epithelial-mesenchymal transition
IL: interleukin
TGF-β: transforming growth factor-beta
MCP-1: monocyte chemoattractant protein-1
PMT: pericyte-to-myofibroblast transition
IRI: ischemia-reperfusion injury
TNF-α: tumor necrosis factor-α
HIF: hypoxia-inducible factor
Wnt: wingless/integrase-1
Shh: sonic hedgehog
Gli1/2: glioma-associated oncogene homolog 1/2
PTCs: peritubular capillaries
CCR2: C-C motif chemokine receptor 2
TLR4: toll-like receptor 4
LPS: lipopolysaccharide
HMGB1: high-mobility group box 1
HSPs: heat shock proteins
AKI: acute kidney injury
C5aR1: C5a receptor 1
NLRP3: NLR family pyrin domain containing 3
MyD88: myeloid differentiation primary response protein 88
CSF1: colony-stimulating factor 1
IFN-γ: interferon-gamma
Th17: T helper 17
Tregs: regulatory T cells
NETs: neutrophil extracellular traps
GSDMD: gasdermin D
ICAM-1: intercellular adhesion molecule 1
TPSB2: tryptase beta 2
FOXD1: Forkhead box D1
EGFR: epidermal growth factor receptor
CTGF: connective tissue growth factor
EV: extracellular vesicle
BMSC: bone marrow mesenchymal stem cell
PI3K: phosphatidylinositol 3-kinase
Akt: protein kinase B
ROCK: rho-associated coiled-coil containing protein kinase
NF-κB: nuclear factor kappa B
ERK1/2: extracellular signal-regulated kinases 1 and 2
TβRI/TβRII: type I/II transforming growth factor-β
JNK: c-Jun N-terminal kinase
TRAF6: TNF receptor-associated factor 6
ShcA: Src homology and collagen A
YAP: Hippo-Yes-associated protein
TAZ: transcriptional co-activator with PDZ-binding motif
MST1/2: mammalian STE20-like kinases 1/2
LATS1/2: large tumor suppressor 1/2
MMP: matrix metalloproteinase
TCF: T-cell factor
LEF: lymphoid enhancer factor
IPF: idiopathic pulmonary fibrosis
SIRT1: sirtuin 1
Dll4: delta-like protein 4
EEVs: engineered extracellular vesicles
GDNF: glial cell line-derived neurotrophic factor
hAMSCs: human adipose mesenchymal stem cells
PLGA: poly(lactic-co-glycolic acid)
FAK: focal adhesion kinase.
